# Uncertainpy: A Python Toolbox for Uncertainty Quantification and Sensitivity Analysis in Computational Neuroscience

**DOI:** 10.3389/fninf.2018.00049

**Published:** 2018-08-14

**Authors:** Simen Tennøe, Geir Halnes, Gaute T. Einevoll

**Affiliations:** ^1^Centre for Integrative Neuroplasticity, University of Oslo, Oslo, Norway; ^2^Department of Informatics, University of Oslo, Oslo, Norway; ^3^Faculty of Science and Technology, Norwegian University of Life Sciences, Ås, Norway; ^4^Department of Physics, University of Oslo, Oslo, Norway

**Keywords:** uncertainty quantification, sensitivity analysis, features, polynomial chaos expansions, quasi-Monte Carlo method, software, computational modeling, Python

## Abstract

Computational models in neuroscience typically contain many parameters that are poorly constrained by experimental data. Uncertainty quantification and sensitivity analysis provide rigorous procedures to quantify how the model output depends on this parameter uncertainty. Unfortunately, the application of such methods is not yet standard within the field of neuroscience. Here we present Uncertainpy, an open-source Python toolbox, tailored to perform uncertainty quantification and sensitivity analysis of neuroscience models. Uncertainpy aims to make it quick and easy to get started with uncertainty analysis, without any need for detailed prior knowledge. The toolbox allows uncertainty quantification and sensitivity analysis to be performed on already existing models without needing to modify the model equations or model implementation. Uncertainpy bases its analysis on polynomial chaos expansions, which are more efficient than the more standard Monte-Carlo based approaches. Uncertainpy is tailored for neuroscience applications by its built-in capability for calculating characteristic features in the model output. The toolbox does not merely perform a point-to-point comparison of the “raw” model output (e.g., membrane voltage traces), but can also calculate the uncertainty and sensitivity of salient model response features such as spike timing, action potential width, average interspike interval, and other features relevant for various neural and neural network models. Uncertainpy comes with several common models and features built in, and including custom models and new features is easy. The aim of the current paper is to present Uncertainpy to the neuroscience community in a user-oriented manner. To demonstrate its broad applicability, we perform an uncertainty quantification and sensitivity analysis of three case studies relevant for neuroscience: the original Hodgkin-Huxley point-neuron model for action potential generation, a multi-compartmental model of a thalamic interneuron implemented in the NEURON simulator, and a sparsely connected recurrent network model implemented in the NEST simulator.

## Significance statement

A major challenge in computational neuroscience is to specify the often large number of parameters that define neuron and neural network models. Many of these parameters have an inherent variability, and some are even actively regulated and change with time. It is important to know how the uncertainty in the model parameters affects the model predictions. To address this need we here present Uncertainpy, an open-source Python toolbox tailored to perform uncertainty quantification and sensitivity analysis of neuroscience models.

## 1. Introduction

Computational modeling has become a useful tool for examining various phenomena in biology in general (Brodland, 2015) and neuroscience in particular (Koch and Segev, 1998; Dayan and Abbott, 2001; Sterratt et al., 2011). The field of neuroscience has seen the development of ever more complex models, and models now exist for large networks of biophysically detailed neurons (Izhikevich and Edelman, 2008; Merolla et al., 2014; Markram et al., 2015).

Computational models typically contain a number of parameters that for various reasons are uncertain. A typical example of an uncertain parameter in a neural model can be the conductance *g*_*x*_ of a fully open ion channel of a specific type *x*. Despite the parameter uncertainty, it is common practice to construct models that are deterministic in the sense that single numerical values are assigned to each parameter.

**Figure 1:**
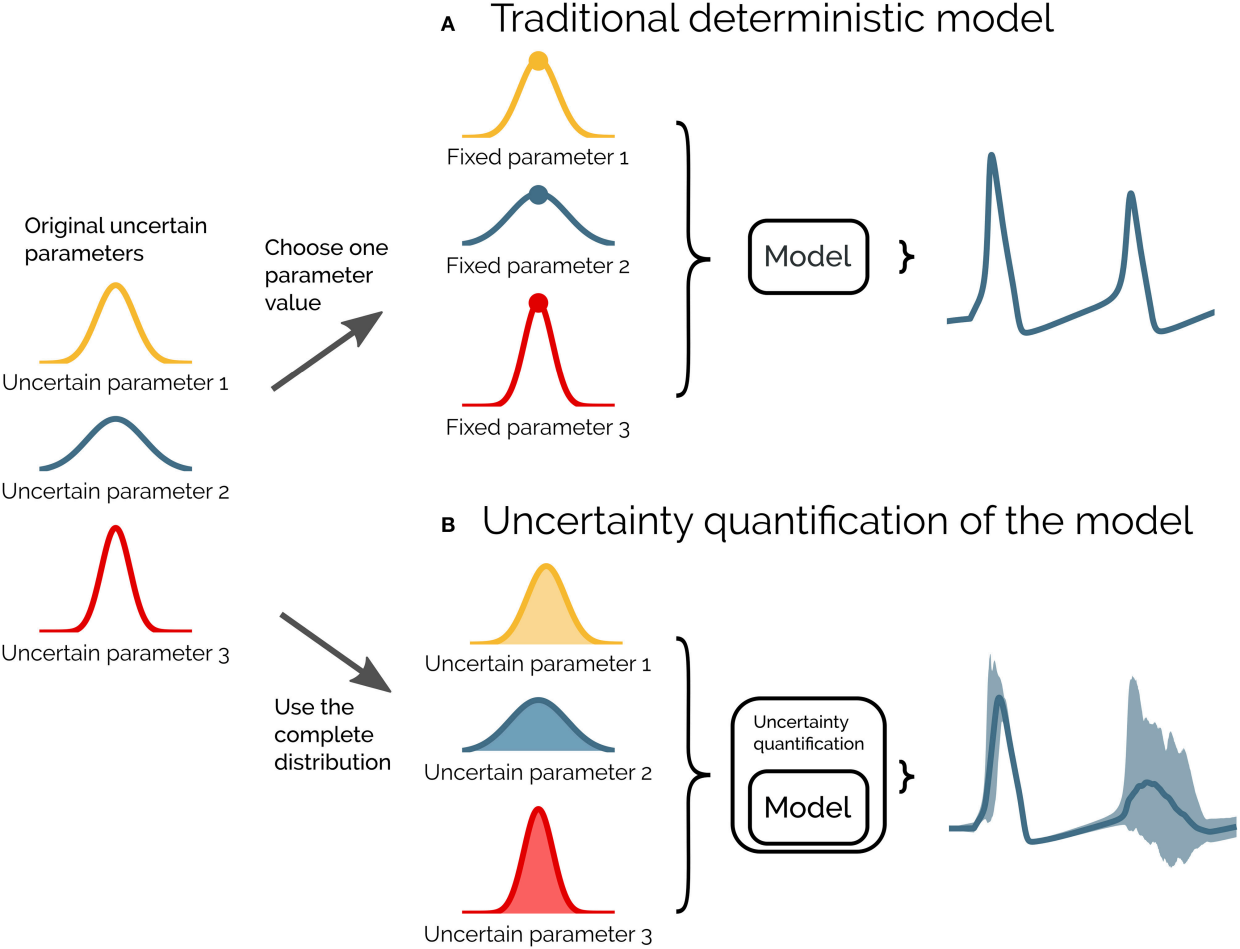
Illustration of uncertainty quantification of a deterministic model. **(A)** A traditional deterministic model where each input parameter has a chosen fixed value, and we get a single output of the model (gray). **(B)** An uncertainty quantification of the model takes the distributions of the input parameters into account, and the output of the model becomes a range of possible values (light gray).

Uncertainty quantification is a means to quantify the uncertainty in the model output that arises from uncertainty in the model parameters. Instead of assuming fixed model parameters as in a deterministic model (as illustrated in Figure 1A), one assigns a distribution of possible values to each model parameter. The uncertainty in the model parameters is then propagated through the model and gives rise to a distribution in the model output (as illustrated in Figure 1B).

Sensitivity analysis is tightly linked to uncertainty quantification and is the process of quantifying how much of the output uncertainty each parameter is responsible for Saltelli (2002b). A small change in a parameter the model is highly sensitive to, leads to a comparatively large change in the model output. Similarly, variations in a parameter the model has a low sensitivity to, result in comparatively small variations in the model output.

Given that most neuroscience models contain a variety of uncertain parameters, the need for systematic approaches to quantify what confidence we can have in the model output is pressing. The importance of uncertainty quantification and sensitivity analysis of computational models is well known in a wide variety of fields (Leamer, 1985; Beck, 1987; Turanyi and Turányi, 1990; Oberkampf et al., 2002; Sharp and Wood-Schultz, 2003; Marino et al., 2008; Najm, 2009; Rossa et al., 2011; Wang and Sheen, 2015; Yildirim and Karniadakis, 2015). Due to the prevalence of inherent variability in the parameters of biological systems, uncertainty quantification and sensitivity analysis are at least as important in neuroscience. Toward this end we have created Uncertainpy^1^, a Python toolbox for uncertainty quantification and sensitivity analysis, tailored toward neuroscience models.

The uncertainty in a model parameter may have many origins. It may be due to (i) measurement uncertainty or (ii) lack of experimental techniques that enable the parameter to be measured. The uncertainty can also be due to an inherent biological variability, meaning the value of a parameter can vary (iii) between neurons of the same species (Edelman and Gally, 2001; Hay et al., 2013), or (iv) dynamically within a single neuron due to plasticity or homeostatic mechanisms (Marder and Goaillard, 2006). Additionally, some models include parameters that are (v) phenomenological abstractions, and therefore do not represent directly measurable physical entities. They might, for example, represent the combined effect of several physical processes. The above uncertainties can generally be divided into two main classes: aleatory uncertainties and epistemic uncertainties. Epistemic uncertainty reflects a lack of knowledge, and can in principle be reduced to zero by acquiring additional information. Aleatory uncertainty, on the other hand, is uncertainty due to inherent variability of the parameters. The importance of distinguishing between aleatory and epistemic uncertainties has evoked some debate (Ferson and Ginzburg, 1996; Hora, 1996; Oberkampf et al., 2002; Ferson et al., 2004; Kiureghian and Ditlevsen, 2009; Mullins et al., 2016), but the distinction is important for how to interpret the results of an uncertainty quantification. Parameters with epistemic uncertainties produce an uncertainty as to whether or not we have acquired the “correct” result, while parameters with aleatory uncertainties reflect the true variability of the system.

A common way to avoid addressing the uncertainty in measured parameters is to use the means of several experimental measurements. This can be problematic since the underlying distribution of a set of parameters can be poorly characterized by the mean and variance of each parameter (Golowasch et al., 2002). Additionally, during model construction, a subset of the uncertain parameters are commonly treated as *free parameters*. This means the parameters are tuned by the modeler to values that make the model output match a set of experimental constraints. An example is fitting an ion-channel conductance *g*_*x*_ so the membrane potential of a neuron model reproduces an experimentally measured voltage trace. Whatever method used, the tuning procedure does not guarantee a unique solution for the correct parameter set, since it is often the case that a wide range of different parameter combinations give rise to similar model behavior (Bhalla and Bower, 1993; Beer et al., 1999; Goldman et al., 2001; Golowasch et al., 2002; Prinz et al., 2004; Tobin, 2006; Halnes et al., 2007; Schulz et al., 2007; Taylor et al., 2009; Marder and Taylor, 2011).

When we have uncertain parameters, but nevertheless choose to use a single set of fixed parameter values, it is a priori difficult to assess to what degree we can trust the model result. Performing an uncertainty quantification enables us to properly take the effects of the uncertain parameters into account, and it quantifies what confidence we can have in the model output. An uncertainty quantification enables us to model the naturally occurring variation in the parameters of biological systems. It also increases our understanding of the model by quantifying how the uncertain parameters influence the model output. Additionally, performing an uncertainty quantification makes comparing two model outputs, as well as a model output and an experimental result, more informative. By knowing the distribution of the model output we can better quantify how similar (or different) the two model outputs, or model output and experimental result, are.

Performing a sensitivity analysis provides insight into how each parameter affects different aspects of the model, and it gives us a better understanding of the relationship between the parameters (and by extent the biological mechanisms) and the output of the model (Marino et al., 2008). A model-based sensitivity analysis can thus help to guide the experimental focus (Zi, 2011). Knowing how sensitive the model is to changes in each parameter, enables us to take special care to obtain accurate measures of parameters with a high sensitivity, while more crude measures are acceptable for parameters with a low sensitivity.

Sensitivity analysis is also useful in model reduction contexts and when performing parameter estimations (Degenring et al., 2004; Zi, 2011; Snowden et al., 2017). A parameter that the model has a low sensitivity to, can essentially be set to any fixed value (within the explored distribution), without greatly affecting the variance of the model output. In some cases, such an analysis can even justify leaving out entire mechanisms from a model. For example, if a single neuron model is insensitive to the conductance of a given ion channel *g*_*x*_, this ion channel could possibly (but not necessarily) be removed from the model with only small changes to the model behavior.

Unfortunately, a generally accepted practice of uncertainty quantification and sensitivity analysis does not currently exist within the field of neuroscience, and models are commonly presented without including any form of uncertainty quantification or sensitivity analysis. When an effort is made in that direction, it is still common to use rather simple, so-called One-At-A-Time methods, where one examines how much the model output changes when varying one parameter at a time (see e.g., De Schutter and Bower, 1994; Blot and Barbour, 2014; Kuchibhotla et al., 2017). Such approaches do not account for potential dependencies between the parameters, and thereby miss correlations within the often multi-dimensional parameter space (Borgonovo and Plischke, 2016). Other methods that have been applied are local methods, which are multi-dimensional, but confined to exploring small perturbations surrounding a single point in the parameter space (see e.g., Gutenkunst et al., 2007; Blomquist et al., 2009; O'Donnell et al., 2017). Such methods can thus not explore the effects of arbitrarily broad uncertainty distributions for the parameters.

Methods for uncertainty quantification and sensitivity analysis that take the entire parameter space into account are often called global methods (Borgonovo and Plischke, 2016; Babtie and Stumpf, 2017). Global methods are only occasionally used within the field of neuroscience (see e.g., Halnes et al., 2009; Torres Valderrama et al., 2015). The most well-known of the global methods is the (quasi-)Monte Carlo method, which relies on randomly sampling the parameter distributions, followed by calculating statistics from the resulting model outputs. The problem with the (quasi-)Monte Carlo method is that it is computationally very demanding, particularly for computationally expensive models. A means to obtain similar results in a much more efficient way, is provided by the recent mathematical framework of polynomial chaos expansions (Xiu and Hesthaven, 2005). Polynomial chaos expansions are used to approximate the model with a polynomial (as a surrogate model), on which the uncertainty and sensitivity analysis can be performed much more efficiently.

To lower the threshold for neuroscientists to perform uncertainty quantification and sensitivity analysis, we have created Uncertainpy, an open-source Python toolbox for efficient uncertainty quantification and sensitivity analysis. Uncertainpy aims to make it quick and easy to get started with uncertainty quantification and sensitivity analysis. Just a few lines of Python code are needed, without any need for detailed prior knowledge of uncertainty or sensitivity analysis. Uncertainpy implements both the quasi-Monte Carlo method and polynomial chaos expansions. The toolbox is model-independent and treats the model as a “black box,” meaning that uncertainty quantification can be performed on already existing models without needing to modify the model equations or model implementation.

Whereas its statistical methods are generally applicable, Uncertainpy is tailored for neuroscience applications by having a built-in capability for recognizing characteristic features in the model output. This means Uncertainpy does not merely perform a point-to-point comparison of the “raw” model output (e.g., a voltage trace). When applicable, Uncertainpy also recognizes and calculates the uncertainty in model response features, for example the spike timing and action-potential shape for neural models and firing rates and interspike intervals for neural networks.

To present Uncertainpy, we start this paper with an overview of the theory behind uncertainty quantification and sensitivity analysis in section 2, with a focus on the (quasi-)Monte Carlo method and polynomial chaos expansions. In section 3 we explain how to use Uncertainpy, and give details on how the uncertainty quantification and sensitivity analysis are implemented. In section 4 we illustrate the use of Uncertainpy by showing four different case studies where we perform uncertainty analysis of: (i) a cooling coffee-cup model (Newton's law of cooling) to illustrate the uncertainty analysis on a conceptually simple model, (ii) the original Hodgkin-Huxley point-neuron model for action potential generation, (iii) a comprehensive multi-compartmental model of a thalamic interneuron, and (iv) a sparsely connected recurrent network model (Brunel network). The final section of section 4 gives a comparison of the performance, that is, numerical efficacy, of the quasi-Monte Carlo method and polynomial chaos expansions using the original Hodgkin-Huxley model as an example. We end with a discussion and some future prospects in section 5.

## 2. Theory on uncertainty quantification and sensitivity analysis

Uncertainty quantification and sensitivity analysis provide rigorous procedures to analyze and characterize the effects of parameter uncertainty on the output of a model. The methods for uncertainty quantification and sensitivity analysis can be divided into global and local methods. Local methods examine how the model output changes with small perturbations around a fixed point in the parameter space. Global methods, on the other hand, take the whole range of parameters into consideration.

The global methods can be divided into intrusive and non-intrusive methods. Intrusive methods require changes to the underlying model equations and are often challenging to implement. Models in neuroscience are often created with the use of advanced simulators such as NEST (Peyser et al., 2017) and NEURON (Hines and Carnevale, 1997). Modifying the underlying equations of models using such simulators is a complicated task best avoided. Non-intrusive methods, on the other hand, consider the model as a black box and can be applied to any model without needing to modify the model equations or model implementation. Global, non-intrusive methods are therefore the methods of choice in Uncertainpy. The uncertainty calculations in Uncertainpy are mainly based on the Python package *Chaospy* (Feinberg and Langtangen, 2015), which provides global, non-intrusive methods for uncertainty quantification and sensitivity analysis. Additionally, Uncertainpy uses the package *SALib* (Herman and Usher, 2017) to perform sensitivity analysis with the quasi-Monte Carlo method.

In this section, we go through the theory behind the methods for uncertainty quantification and sensitivity analysis used in Uncertainpy. We start by introducing the notation used in this paper (section 2.1). Next, we introduce the statistical measurements for uncertainty quantification (section 2.2) and sensitivity analysis (section 2.3). Further, we give an introduction to the (quasi-)Monte Carlo method (section 2.4) and polynomial chaos expansions (section 2.5), the two methods used to perform the uncertainty analysis in Uncertainpy. We next explain how Uncertainpy handles cases with statistically dependent model parameters (section 2.6). Finally, we explain the concept and benefits of performing a feature-based analysis (section 2.7). We note that detailed insight into the theory of uncertainty quantification and sensitivity analysis is not a prerequisite for *using* Uncertainpy, so the more practically oriented reader may choose to skip this section, and go directly to the user guide in section 3.

### 2.1. Problem definition

Consider a model *U* that depends on space ***x*** and time *t*, has *d* uncertain input parameters ***Q*** = [*Q*_1_, *Q*_2_, …, *Q*_*d*_], and gives the output *Y*:

(1) $$\begin{array}{l} Y=U\left(\text{x},t,\text{Q}\right). \end{array}$$

The output *Y* can have any value within the output space Ω_*Y*_ and has an unknown probability density function ρ_*Y*_. The goal of an uncertainty quantification is to describe the unknown ρ_*Y*_ through statistical metrics. We are only interested in the input and output of the model, and we ignore all details on the inner workings of the model. The model *U* is thus considered a black box and may represent any model, for example a spiking neuron model that returns a voltage trace, or a neural network model that returns a spike train.

We assume the model includes uncertain parameters that can be described by a multivariate probability density function ρ_***Q***_. Examples of parameters that can be uncertain in neuroscience are the conductance of a single ion channel or the synaptic weight between two types of neurons in a neural network. If the uncertain parameters are statistically independent, the multivariate probability density function ρ_***Q***_ can be given as separate univariate probability density functions ρ_*Q*_*i*__, one for each uncertain parameter *Q*_*i*_. The joint multivariate probability density function for the independent uncertain parameters is then:

(2) $$\begin{array}{l} \rho_{\text{Q}}=\overset{d}{\underset{i=1}{\prod }}\rho_{Q_{i}}. \end{array}$$

In cases where the uncertain input parameters are statistically dependent variables, the multivariate probability density function ρ_***Q***_ must be defined directly. It should be noted that with statistically dependent parameters we here mean that there is a dependence between the input parameters. When drawing parameters from the joint probability function, by drawing one parameter we influence the probability of drawing specific values for the other parameters. Thus, we do not refer to dependencies between how the input parameters affect the model *output*. We assume the probability density functions are known and are not here concerned with how they are determined. They may be the product of a series of measurements, a parameter estimation, or educated guesses.

### 2.2. Uncertainty quantification

As mentioned, the goal of an uncertainty quantification is to describe the unknown distribution of the model output ρ_*Y*_ through statistical metrics. The two most common statistical metrics used in this context are the mean 𝔼 (also called the expectation value) and the variance 𝕍. The mean is defined as:

(3) $$\begin{array}{l} 𝔼\left[Y\right]=\int_{\Omega_{Y}}y\rho_{Y}\left(y\right)dy, \end{array}$$

and tells us the expected value of the model output *Y*. The variance is defined as:

(4) $$\begin{array}{l} 𝕍\left[Y\right]=\underset{\Omega_{Y}}{\int }{\left(y-𝔼\left[Y\right]\right)}^{2}\rho_{Y}\left(y\right)dy, \end{array}$$

and tells us how much the output varies around the mean.

Another useful metric is the (100·*x*)-th percentile *P*_*x*_ of *Y*, which defines a value below which 100·*x* percent of the model outputs are located. For example, 5% of the evaluations of a model will give an output lower than the 5th percentile. The (100 · *x*)-th percentile is defined as:

(5) $$\begin{array}{l} x=\int_{-\infty }^{P_{x}}\rho_{Y}\left(y\right)dy. \end{array}$$

We can combine two percentiles to create a prediction interval *I*_*x*_, which is a range of values within which a 100 · *x* percentage of the outputs *Y* occur:

(6) $$\begin{array}{l} I_{x}=\left[P_{\left(x/2\right)},P_{\left(1-x/2\right)}\right]. \end{array}$$

The 90% prediction interval gives us the interval within which 90% of the *Y* outcomes occur, which also means that 5% of the outcomes are above and 5% are below this interval.

### 2.3. Sensitivity analysis

A sensitivity analysis quantifies how much of the uncertainty in the model output each uncertain parameter is responsible for. Several different sensitivity measures exist, for a review of methods for sensitivity analysis see Saltelli et al. (2007), Hamby (1994), and Zi (2011). Uncertainpy uses variance-based sensitivity analysis and computes the commonly considered Sobol sensitivity indices (Sobol, 1990). This sensitivity analysis is global, non-intrusive and allows the effects of interactions between parameters within the model to be studied (Zi, 2011). (Two parameters are said to interact if they have a non-additive effect on the output (Saltelli et al., 2007).)

The Sobol sensitivity indices quantify how much of the variance in the model output each uncertain parameter is responsible for. If a parameter has a low sensitivity index, variations in this parameter result in comparatively small variations in the final model output. Similarly, if a parameter has a high sensitivity index, a change in this parameter leads to a large change in the model output.

There are several types of Sobol indices. The first-order Sobol sensitivity index *S*_*i*_ measures the direct effect each parameter has on the variance of the model:

(7) $$\begin{array}{l} S_{i}=\frac{𝕍\left[𝔼\left[Y|Q_{i}\right]\right]}{𝕍\left[Y\right]}. \end{array}$$

Here, 𝔼[*Y*|*Q*_*i*_] denotes the expected value of the output *Y* when the parameter *Q*_*i*_ is fixed. The first-order Sobol sensitivity index tells us the expected reduction in the variance of the model when we fix parameter *Q*_*i*_. The sum of the first-order Sobol sensitivity indices cannot exceed one, and is only equal to one if no interactions are present (Glen and Isaacs, 2012).

Higher order Sobol indices exist and give the sensitivity due to interactions between a parameter *Q*_*i*_ and various other parameters. It is customary to only calculate the first and total-order indices (Saltelli et al., 2010). The total Sobol sensitivity index *S*_*Ti*_ includes the sensitivity of both the first-order effects, as well as the sensitivity due to interactions between a given parameter *Q*_*i*_ and all combinations of the other parameters (Homma and Saltelli, 1996). It is defined as:

(8) $$\begin{array}{l} S_{Ti}=1-\frac{𝕍\left[𝔼\left[Y|Q_{-i}\right]\right]}{𝕍\left[Y\right]}, \end{array}$$

where *Q*_−*i*_ denotes all uncertain parameters except *Q*_*i*_. The sum of the total Sobol sensitivity indices is equal to or greater than one, and is only equal to one if there are no interactions between the parameters (Glen and Isaacs, 2012). When the goal is to use sensitivity analysis to fix parameters with low sensitivity, it is recommended to use the total-order Sobol indices.

We might want to compare Sobol indices across different features (introduced in section 2.7). This can be problematic when we have features with a different number of output dimensions. In the case of a zero-dimensional output, the Sobol indices are a single number and for a one-dimensional output we get Sobol indices for each point in time. To better be able to compare the Sobol indices across such features, we also calculate the average of the first-order Sobol indices ${\bar{S}}_{i}$, and total-order Sobol indices ${\bar{S}}_{Ti}$.

### 2.4. (Quasi-)Monte Carlo method

A typical way to obtain the statistical metrics mentioned above is to use the (quasi-)Monte Carlo method. We give a brief overview of the Monte Carlo and quasi-Monte Carlo method here, for a more comprehensive review see Lemieux (2009).

The general idea behind the standard Monte Carlo method is quite simple. A set of parameters is randomly drawn from the joint multivariate probability density function ρ_**Q**_ of the parameters. The model is then evaluated for the sampled parameter set. This process is repeated thousands of times, and statistical metrics such as the mean and variance are computed from the resulting series of model outputs. The accuracy of the Monte Carlo method, and by extent the number of samples required, is independent of the number of uncertain parameters. Additionally, the Monte Carlo method makes no assumptions about the model. However, a limitation of the Monte Carlo method is that a very high number of model evaluations are required to get reliable statistics. If the model is computationally expensive, the Monte Carlo method may thus require insurmountable computer power.

The quasi-Monte Carlo method improves upon the standard Monte Carlo method by using variance-reduction techniques to reduce the number of model evaluations needed. This method is based on increasing the coverage of the sampled parameter space by distributing the samples more evenly. Fewer samples are then required to obtain a given accuracy. Instead of randomly selecting parameters from ρ_***Q***_, the samples are selected using a low-discrepancy sequence such as the Sobol sequence or Hammersley sequence (Hammersley, 1960; Sobol, 1967). The quasi-Monte Carlo method is faster than the Monte Carlo method, as long as the number of uncertain parameters is sufficiently small, and the model is sufficiently smooth (Lemieux, 2009).

Uncertainpy allows the quasi-Monte Carlo method to be used to compute the statistical metrics. When this option is chosen, the metrics are computed as follows. With *N*_*s*_ model evaluations, which gives the results ***Y*** = [*Y*_1_, *Y*_2_, …, *Y*_*N*_*s*__], the mean is given by

(9) $$\begin{array}{l} 𝔼\left[Y\right]\approx \frac{1}{N_{s}}\overset{N_{s}}{\underset{i=1}{\sum }}Y_{i}, \end{array}$$

and the variance by

(10) $$\begin{array}{l} 𝕍\left[Y\right]\approx \frac{1}{N_{s}-1}\overset{N_{s}}{\underset{i=1}{\sum }}{\left(Y_{i}-𝔼\left[Y\right]\right)}^{2}. \end{array}$$

Prediction intervals are found by sorting the model evaluations ***Y*** in an ascending order, and then finding the (100 · *x*/2)-th and (100 · (1 − *x*/2))-th percentiles. The Sobol indices can be calculated using Saltelli's method (Saltelli, 2002a; Saltelli et al., 2010). The number of samples required by this method is:

(11) $$\begin{array}{l} N_{s}=N\left(d+2\right), \end{array}$$

where *N* is the number of samples required to get a given accuracy with the quasi-Monte Carlo method. This means that the number of samples required by both the Monte Carlo method and the quasi-Monte Carlo method for sensitivity analysis depends on the number of uncertain parameters. Due to how the samples are selected in Saltelli's method, when selecting *N* samples for the uncertainty quantification (which give *N*_*s*_ = *N*), we get *N*_*s*_ = *N*(*d* + 2)/2 samples for the sensitivity analysis. The chosen number of samples *N* is effectively halved.

It should be noted that there is no guarantee that each set of sampled parameters will produce a valid model evaluation. For example, the spike width will not be defined for a model that produces no spikes. The (quasi-)Monte Carlo method is robust for such missing model results when performing an uncertainty quantification, as long as the number of valid model evaluations is relatively high. However, for the sensitivity analysis the (quasi-) Monte Carlo method using Saltelli's approach requires that there are no missing model results. A suggested workaround (Herman and Usher, 2017) is to replace invalid model evaluations with the mean of the evaluations^2^. This workaround introduces an error depending on the number of missing evaluations but enables us to still calculate the Sobol indices. This workaround is used in Uncertainpy.

### 2.5. Polynomial chaos expansions

A recent mathematical framework for efficient uncertainty quantification and sensitivity analysis is that of polynomial chaos expansions (Xiu and Hesthaven, 2005). This method calculates the same statistical metrics as the (quasi-)Monte Carlo method but is typically much faster (Xiu and Hesthaven, 2005; Crestaux et al., 2009; Eck et al., 2016). For the Hodgkin-Huxley model, we find that polynomial chaos expansions require one to three orders of magnitude fewer model evaluations than the quasi-Monte Carlo method (see section 4.5). We here give a short review of polynomial chaos expansions, for a comprehensive review see Xiu (2010).

Polynomial chaos expansions are typically much faster than the (quasi-)Monte Carlo method as long as the number of uncertain parameters is relatively low, typically smaller than about 20 (Xiu and Hesthaven, 2005; Crestaux et al., 2009; Eck et al., 2016). This means polynomial chaos expansions require far fewer model evaluations than the (quasi-)Monte Carlo method to obtain the same accuracy. It is often the case that neuroscience models have fewer than about 20 parameters, and even for models with a higher number of uncertain parameters, polynomial chaos expansions can be used for selected subsets of the parameters.

The main limitation of polynomial chaos expansions is that the required number of model evaluations scales worse with an increasing number of uncertain parameters than the (quasi-)Monte Carlo method does. This is the reason why the (quasi-)Monte Carlo method becomes better at around 20 uncertain parameters. Another limitation of the polynomial chaos expansions is that the performance is reduced if the output has a non-smooth behavior with respect to the input parameters (Eck et al., 2016).

The exact gain in efficiency when using polynomial chaos expansions instead of the quasi-Monte Carlo method is problem dependent. However, Crestaux et al. (2009) examined three different benchmark problems with three, twelve, and five uncertain parameters. They found that the error in the polynomial chaos expansions converged as $N_{s}^{-6}$, $N_{s}^{-2}$, and between $N_{s}^{-1}$ and $N_{s}^{-3/4}$, respectively. In comparison, the error of the quasi-Monte Carlo method converged as $\sim N_{s}^{-3/4}$ for each of the problems. Polynomial chaos expansions thus have a much faster convergence for the first two benchmark problems, while the convergences were essentially the same for the last problem. The last benchmark problem was non-smooth, which led to the slower convergence of the polynomial chaos expansions. Still, even in the worst-case example considered in Crestaux et al. (2009), the convergence of the polynomial chaos expansions was essentially as good as for the quasi-Monte Carlo method.

The general idea behind polynomial chaos expansions is to approximate the model *U* with a polynomial expansion Û:

(12) $$\begin{array}{l} U\approx Û\left(\text{x},t,\text{Q}\right)=\overset{N_{p}-1}{\underset{n=0}{\sum }}c_{n}\left(\text{x},t\right)\phi_{n}\left(\text{Q}\right), \end{array}$$

where **ϕ**_*n*_ are polynomials, and *c*_*n*_ are expansion coefficients. The number of expansion factors *N*_*p*_ is given by

(13) $$\begin{array}{l} N_{p}=\left(\begin{array}{c} d+p\\ p \end{array}\right), \end{array}$$

where *p* is the polynomial order. The polynomials ϕ_*n*_(***Q***) are chosen so they are orthogonal with respect to the probability density function ρ_***Q***_, which ensures useful statistical properties.

When creating the polynomial chaos expansion, the first step is to find the orthogonal polynomials **ϕ**_*n*_. In Uncertainpy this is done using the so-called three-term recurrence relation (Xiu, 2010) if available, otherwise the discretized Stieltjes method (Stieltjes, 1884) is used. The next step is to estimate the expansion coefficients *c*_*n*_. The non-intrusive methods for doing this can be divided into two classes, point-collocation methods and pseudo-spectral projection methods, both of which are implemented in Uncertainpy.

Point collocation is the default method used in Uncertainpy. This method is based on demanding that the polynomial approximation is equal to the model output evaluated at a set of collocation nodes drawn from the joint probability density function ρ_***Q***_. This demand results in a set of linear equations for the polynomial coefficients *c*_*n*_, which can be solved by the use of regression methods. The regression method used in Uncertainpy is Tikhonov regularization (Rifkin and Lippert, 2007). Hosder et al. (2007) recommends using *N*_*s*_ = 2(*N*_*p*_ + 1) collocation nodes.

Pseudo-spectral projection methods are based on least squares minimization in the orthogonal polynomial space and calculate the expansion coefficients *c*_*n*_ through numerical integration. The integration uses a quadrature scheme with weights and nodes, and the model is evaluated at these nodes. The number of samples is determined by the quadrature rule. The quadrature method used in Uncertainpy is Leja quadrature, with Smolyak sparse grids to reduce the number of required nodes (Smolyak, 1963; Narayan and Jakeman, 2014). Pseudo-spectral projection is only used in Uncertainpy when requested by the user.

Of these two methods, point collocation is robust toward invalid model evaluations as long as the number of remaining evaluations is high enough, while spectral projection is not (Eck et al., 2016).

Several of the statistical metrics of interest can be obtained directly from the polynomial chaos expansion Û. The mean is simply

(14) $$\begin{array}{l} 𝔼\left[Y\right]\approx c_{0}, \end{array}$$

and the variance is

(15) $$\begin{array}{l} 𝕍\left[Y\right]\approx \overset{N_{p}-1}{\underset{n=1}{\sum }}\gamma_{n}c_{n}^{2}, \end{array}$$

where γ_*n*_ is a normalization factor defined as

(16) $$\begin{array}{l} \gamma_{n}=𝔼\left[\phi_{n}^{2}\left(\text{Q}\right)\right]. \end{array}$$

The first and total-order Sobol indices can also be calculated directly from the polynomial chaos expansion (Sudret, 2008; Crestaux et al., 2009). On the other hand, the percentiles (Equation 5), and thereby the prediction interval (Equation 6), must be estimated by using Û as a surrogate model and then performing the same procedure as for the (quasi-)Monte Carlo method.

### 2.6. Dependency between uncertain parameters

One of the underlying assumptions when creating the polynomial chaos expansions is that the model parameters are independent. However, dependent parameters in neuroscience models are quite common (Achard and De Schutter, 2006). Fortunately, models containing dependent parameters can be analyzed with Uncertainpy by the aid of the Rosenblatt transformation from Chaospy (Rosenblatt, 1952; Feinberg and Langtangen, 2015). Briefly explained, the idea is to create a reformulated model $\tilde{U}\left(\text{x},t,\text{R}\right)$ based on an independent parameter set ***R***, and then perform polynomial chaos expansions on the reformulated model. The Rosenblatt transformation is used to construct the reformulated model so it gives the same output (and statistics) as the original model, i.e.,:

(17) $$\begin{array}{l} \tilde{U}\left(\text{x},t,\text{R}\right)=U\left(\text{x},t,\text{Q}\right). \end{array}$$

For more information on the use of the Rosenblatt transformation, see the Uncertainpy documentation^3^ or Feinberg and Langtangen (2015).

### 2.7. Feature-based analysis

When measuring the membrane potential of a neuron, the precise timing of action potentials often varies between recordings, even if the experimental conditions are the same. This behavior is typical for biological systems. Since the experimental data displays such variation, it is often meaningless and misleading to base the success of a computational model on a direct point-to-point comparison between a particular experimental recording and model output (Druckmann et al., 2007; Van Geit et al., 2008). A common modeling practice is therefore to have the model reproduce essential features of the experimentally observed dynamics, such as the action-potential shape or action-potential firing rate (Druckmann et al., 2007). Such features are typically more robust across different experimental measurements, or across different model simulations, than the raw data or raw model output itself, at least if sensible features have been chosen.

Uncertainpy takes this aspect of neural modeling into account and is constructed so that it can extract a set of features relevant for various common model types in neuroscience from the raw model output. Examples include the action potential shape in single neuron models and the average interspike interval in neural network models. Thus Uncertainpy performs an uncertainty quantification and sensitivity analysis not only on the raw model output but also on a set of relevant features selected by the user. Lists of the implemented features are given in section 3.4, and the value of a feature-based analysis is illustrated in two of the case studies (sections 5.3 and 5.4).

## 3. User guide for uncertainpy

Uncertainpy is a Python toolbox, tailored to make uncertainty quantification and sensitivity analysis easily accessible to the computational neuroscience community. The toolbox is based on Python, since Python is a high level, open-source language in extensive and increasing use within the scientific community (Oliphant, 2007; Einevoll, 2009; Muller et al., 2015). Uncertainpy works with both Python 2 and 3, and utilizes the Python packages Chaospy (Feinberg and Langtangen, 2015) and SALib (Herman and Usher, 2017) to perform the uncertainty calculations. In this section, we present a guide on to how to use Uncertainpy. We do not present an exhaustive overview, and only show the most commonly used classes, methods and method arguments. We refer to the online documentation^4^ for the most recent, complete documentation. A complete case study with code is shown in section 4.1.

Uncertainpy is easily installed by following the instructions in section 3.8. After installation, we get access to Uncertainpy by simply importing it:


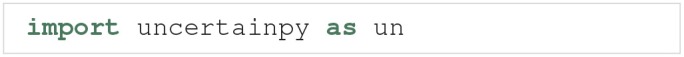


Performing an uncertainty quantification and sensitivity analysis with Uncertainpy includes three main components:

1. The **model** we want to examine.
2. The **parameters** of the model.
3. Specifications of **features** in the model output.

The model and parameters are required components, while the feature specifications are optional. The three (or two) components are brought together in the UncertaintyQuantification class. This class performs the uncertainty calculations and is the main class the user interacts with. In this section, we explain how to use UncertaintyQuantification with the above components, and introduce a few additional utility classes.

### 3.1. The uncertainty quantification class

The UncertaintyQuantification class is used to define the problem, perform the uncertainty quantification and sensitivity analysis, and save and visualize the results. Among others, UncertaintyQuantification takes the arguments:


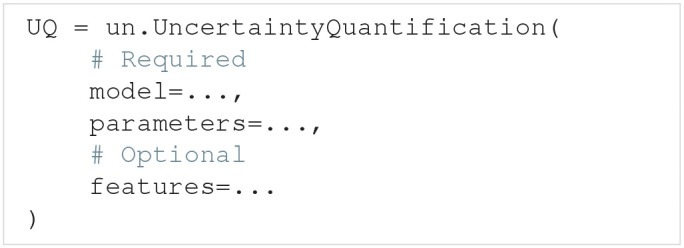


The model argument is either a Model instance (section 3.2) or a model function (section 3.2.2). The parameters argument is either a Parameters instance or a parameter dictionary (section 3.3). Lastly, the features argument is either a Features instance (section 3.4) or a list of feature functions (section 3.4.1). In general, using the class instances as arguments give more options, while using the corresponding functions are slightly easier. We go through how to use each of these classes and corresponding functions in the next three sections.

After the problem is set up, an uncertainty quantification and sensitivity analysis can be performed by using the UncertaintyQuantification.quantify method. Among others, quantify takes the optional arguments:


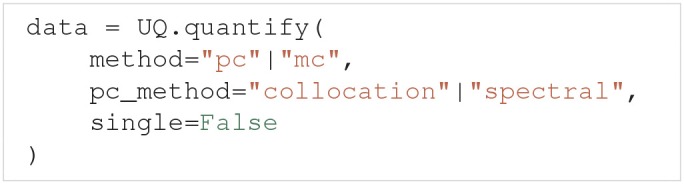


The method argument allows the user to choose whether Uncertainpy should use polynomial chaos expansions ("pc") or the quasi-Monte Carlo method ("mc") to calculate the relevant statistical metrics. If polynomial chaos expansions are chosen, pc_method further specifies whether point collocation ("collocation") or spectral projection ("spectral") methods are used to calculate the expansion coefficients. single specifies whether we perform the uncertainty quantification for a single parameter at the time, or consider all uncertain parameters at once. Performing the uncertainty quantification for one parameter at the time is a simple form of screening. The idea of such a screening is to use a computationally cheap method to reduce the number of uncertain parameters by setting the parameters that have the least effect on the model output to fixed values. We can then consider only the parameters with the greatest effect on the model output when performing the “full” uncertainty quantification and sensitivity analysis. This screening can be performed using both polynomial chaos expansions and the quasi-Monte Carlo method, but polynomial chaos expansions are almost always the faster choice. If nothing is specified, Uncertainpy by default uses polynomial chaos expansions based on point collocation with all uncertain parameters. The Rosenblatt transformation is automatically used if the input parameters are dependent.

The results from the uncertainty quantification are returned in data, as a Data object (see section 3.6). By default, the results are also automatically saved in a folder named data, and the figures are automatically plotted and saved in a folder named figures, both in the current directory. The returned Data object is therefore not necessarily needed.

As mentioned earlier, there is no guarantee that each set of sampled parameters produces a valid model or feature output. In such cases, Uncertainpy gives a warning which includes the number of runs that failed to return a valid output and performs the uncertainty quantification and sensitivity analysis using the reduced set of valid runs. However, if a large fraction of the simulations fail, the user could consider redefining the problem (e.g., by using narrower parameter distributions).

Polynomial chaos expansions are recommended as long as the number of uncertain parameters is small (typically < 20), as polynomial chaos expansions in these cases are much faster than the quasi-Monte Carlo method. Which of the polynomial chaos expansion methods to preferably use is problem dependent. In general, the pseudo-spectral method is faster than point collocation, but has a lower stability. We therefore recommend to use the point-collocation method.

The accuracy of the quasi-Monte Carlo method and polynomial chaos expansions is problem dependent and is determined by the chosen number of samples *N*, as well as the polynomial order *p* for polynomial chaos expansions. It is therefore a good practice to examine if the results from the uncertainty quantification and sensitivity analysis have converged (Eck et al., 2016). A simple method for doing this is to increase or decrease the number of samples or polynomial order, or both, and examine the difference between the current and previous results. If the differences are small enough, we can be reasonably certain that we have an accurate result.

### 3.2. Models

In order to perform the uncertainty quantification and sensitivity analysis of a model, Uncertainpy needs to set the parameters of the model, run the model using those parameters, and receive the model output. Uncertainpy has built-in support for NEURON and NEST models, found in the NeuronModel (section 3.2.4) and NestModel (section 3.2.5) classes respectively. It should be noted that while Uncertainpy is tailored toward neuroscience, it is not restricted to neuroscience models. Uncertainpy can be used on any model that meets the criteria in this section. Below, we first explain how to create custom models, before we explain how to use NeuronModel and NestModel.

#### 3.2.1. The model class

Generally, models are created through the Model class. Among others, Model takes the argument run and the optional arguments interpolate, labels, postprocess and ignore.


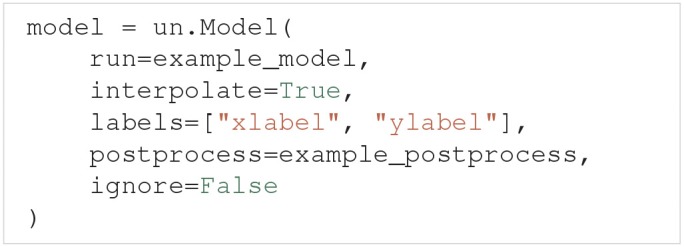


The run argument must be a Python function that runs a simulation on a specific model for a given set of model parameters and returns the simulation output. In this paper we call such a function a model function. If we set interpolate=True, Uncertainpy automatically interpolates the model output to a regular form, meaning each model evaluation has the same number of measurement points (most commonly time points). An irregular model, on the other hand, has a varying number of measurement points between different evaluations (the output is on an irregular form), a typical example is a model that uses adaptive time steps. The uncertainty quantification requires the model output to be on a regular form, and we must set interpolate=True for irregular models. labels allows the user to specify a list of labels to be used on the axes when plotting the results. The postprocess argument is a Python function used to post-process the model output if required. We will go into details on the requirements of the postprocess and model functions below. Finally, if ignore=True we do not perform an uncertainty quantification of the model output. This is used if we want to examine features of the model, but are not interested in the model result itself.

#### 3.2.2. Defining a model function

As explained above, the run argument is a Python function that runs a simulation of a specific model for a given set of model parameters, and returns the simulation output. An example outline of a model function is:


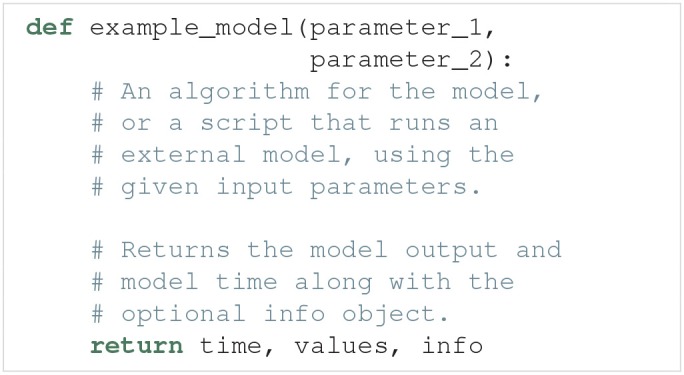


Such a model function has the following requirements:

1. **Input**. The model function takes a number of arguments which define the uncertain parameters of the model.
2. **Run the model**. The model must then be run using the parameters given as arguments.
3. **Output**. The model function must return at least two objects, the model time (or equivalent, if applicable) and model output. Additionally, any number of optional info objects can be returned. In Uncertainpy, we refer to the time object as time, the model output object as values, and the remaining objects as info.
  - **Time** (time). time can be interpreted as the *x*-axis of the model. It is used when interpolating (see below), and when certain features are calculated. We can return None if the model has no time associated with it.
  - **Model output** (values). The model output must either be regular (each model evaluation has the same number of measurement points), or it must be possible to interpolate or post-process the output (see section 3.2.3) to a regular form.
  - **Additional info** (info). Some of the methods provided by Uncertainpy, such as the later defined model post-processing, feature pre-processing, and feature calculations, require additional information from the model (e.g., the time when a neuron receives an external stimulus). This information can be passed on as any number of additional info objects returned after time and values. We recommend using a single dictionary as info object, with key-value pairs for the information, to make debugging easier. Uncertainpy always uses a single dictionary as the info object. Certain features require specific keys to be present in this dictionary.

The model itself does not need to be implemented in Python. Any simulator can be used, as long as we can set the model parameters and retrieve the simulation output via Python. As a shortcut, we can pass a model function to the model argument in UncertaintyQuantification, instead of first having to create a Model instance.

#### 3.2.3. Defining a post-process function

**Figure 2:**
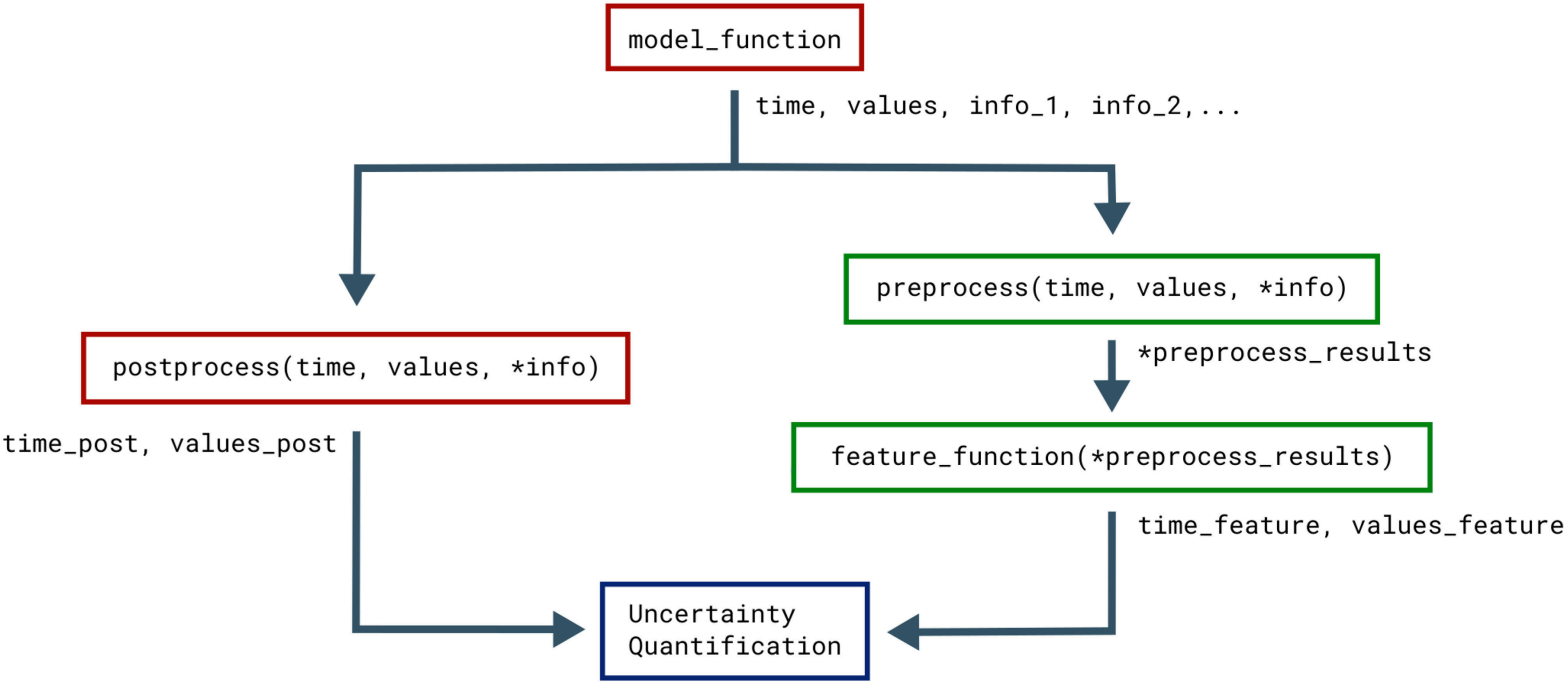
Classes that affect the objects returned by the model. The Uncertainpy methods that use, change, and perform calculations on the objects returned by the model function (time, values, and the optional info). Functions associated with the model are in red while functions associated with features are in green.

The postprocess function is used to post-process the model output before it is used in the uncertainty quantification. Post-processing does not change the model output sent to the feature calculations. This is useful if we need to transform the model output to a regular form for the uncertainty quantification, but still need to preserve the original model output to reliably detect the model features. Figure 2 illustrates how the objects returned by the model function are sent to both model postprocess and feature preprocess (see section 3.4).

An example outline of the postprocess function is:


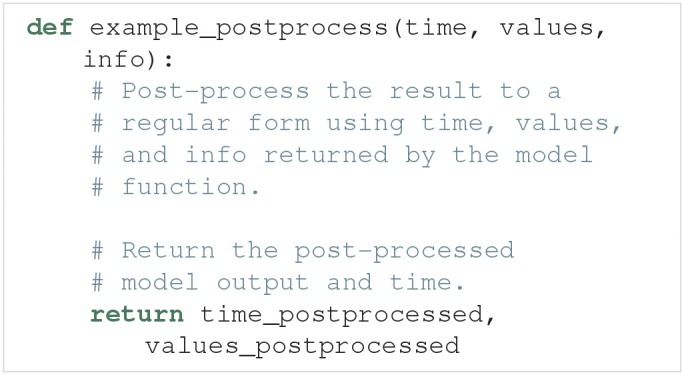


The only time post-processing is required for Uncertainpy to work is when the model produces output that cannot be interpolated to a regular form by Uncertainpy. Post-processing is for example required for network models that give output in the form of spike trains, i.e., time values indicating when a given neuron fires. It should be noted that post-processing of spike trains is already implemented in Uncertainpy (see section 3.2.5). For most purposes, user-defined post-processing will not be necessary.

The requirements for the postprocess function are:

1. **Input**. The postprocess function must take the objects returned by the model function as input arguments.
2. **Post-processing**. The model time (time) and output (values) must be post-processed to a regular form, or to a form that can be interpolated to a regular form by Uncertainpy. If additional information is needed from the model, it can be passed along in the info object.
3. **Output**. The postprocess function must return two objects:
  - **Model time** (time_postprocessed). The first object is the post-processed time (or equivalent) of the model. We can return None if the model has no time. Note that the automatic interpolation can only be performed if a post-processed time is returned (if an interpolation is required).
  - **Model output** (values_postprocessed). The second object is the post-processed model output.

#### 3.2.4. NEURON model class

NEURON (Hines and Carnevale, 1997) is a widely used simulator for multi-compartmental neural models. Uncertainpy has support for NEURON models through the NeuronModel class, a subclass of Model. Among others, NeuronModel takes the arguments:


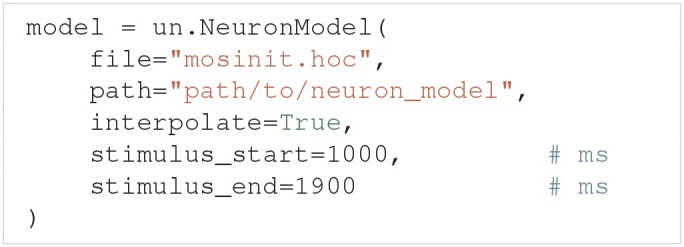


The file argument is the name of the hoc file that loads the NEURON model, which by default is mosinit.hoc. path is the path to the folder where the NEURON model is saved (the location of the mosinit.hoc file). interpolate indicates whether the NEURON model uses adaptive time steps and therefore should be interpolated. stimulus_start and stimulus_end denote the start and end time of any stimulus given to the neuron. NeuronModel loads the NEURON model from file, sets the parameters of the model, evaluates the model and returns the somatic membrane potential of the neuron (we record the voltage from the segment named "soma"). NeuronModel therefore does not require a model function to be defined. A case study of a NEURON model analyzed with Uncertainpy is found in section 4.3.

If changes are needed to the standard NeuronModel, such as measuring the voltage from other locations than the soma, the Model class with an appropriate model function could be used instead. Alternatively, NeuronModel can be subclassed and the existing methods customized as required. An example of the latter is shown in uncertainpy/examples/bahl/.

#### 3.2.5. NEST model class

NEST (Peyser et al., 2017) is a simulator for large networks of spiking neurons. NEST models are supported through the NestModel class, another subclass of Model:


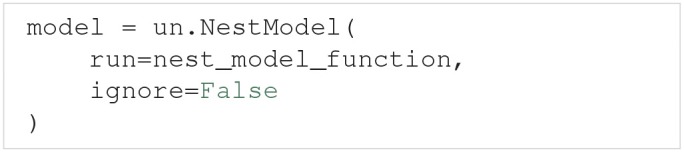


Unlike NeuronModel, NestModel requires the model function to be specified through the run argument. The NEST model function has the same requirements as a regular model function, except it is restricted to return only two objects: the final simulation time (denoted simulation_end), and a list of spike times for selected neurons in the network, which we refer to as spike trains (denoted spiketrains).

A spike train returned by a NEST model is a set of irregularly spaced time points where a neuron fired a spike. NEST models therefore require post-processing to make the model output regular. Such a post-processing is provided by the implemented NestModel.postprocess method, which converts a spike train to a list of zeros (no spike) and ones (a spike) for each time step in the simulation. For example: If a NEST simulation returns the spike train [0, 2, 3.5], it means the neuron fired three spikes occurring at *t* = 0, 2, and3.5 ms, respectively. If the simulation has a time resolution of 0.5 ms and ends after 4 ms, NestModel.postprocess will return the post-processed spike train [1, 0, 0, 0, 1, 0, 0, 1, 0], and the post-processed time array [0, 0.5, 1, 1.5, 2, 2.5, 3, 3.5, 4]. The final uncertainty quantification of a NEST network therefore predicts the probability for a spike to occur at any specific time point in the simulation. It should be noted that performing an uncertainty quantification of the post-processed NEST model output is computationally expensive. As such we recommend setting ignore=ignore=True as long as you are not interested in the uncertainty of the spike trains from the network. An Uncertainpy-based analysis of a NEST model is found in the case study in section 4.4.

### 3.3. Parameters of the model

The parameters of a model are defined by two properties: They must have (i) a name and (ii) either a fixed value or a distribution. It is important that the name of a parameter is the same as the name given as the input argument in the model function. A parameter is considered uncertain if it is given a probability distribution, which are defined using Chaospy. 64 different univariate distributions are available in Chaospy, and Chaospy has support for easy creation of multivariate distributions. For a list of available distributions and detailed instructions on how to create probability distributions with Chaospy, see section 3.3 in Feinberg and Langtangen (2015).

The parameters are defined by the Parameters class. Parameters takes the argument parameters, which is a dictionary where the names of the parameters are the keys, and the fixed values or distributions of the parameters are the values. Here is an example of such a parameter dictionary with two parameters, where the first is named name_1 and has a uniform probability distribution in the interval [8, 16], and the second is named name_2 and has a fixed value of 42:


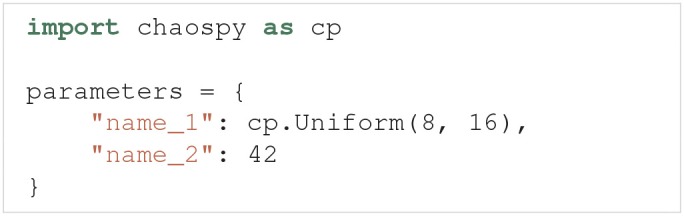


Parameters is now initialized as:


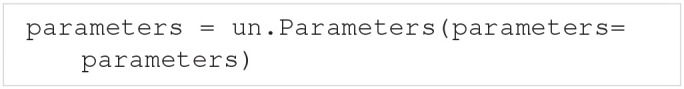


As a shortcut, we can pass the above parameter dictionary to the parameters argument in UncertaintyQuantification, instead of first having to create a Parameters instance.

If the parameters do not have separate univariate probability distributions, but a joint multivariate probability distribution, the multivariate distribution can be set by giving Parameters the optional argument distribution:


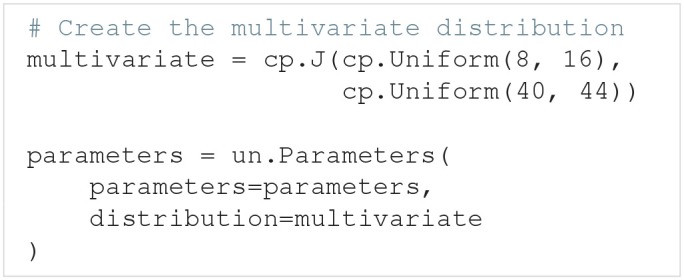


### 3.4. Features

As discussed in section 2.7, it is often more meaningful to examine the uncertainty in salient features of the model output, than to base the analysis directly on a point-to-point comparison of the raw output data (e.g., a voltage trace). Upon user request, Uncertainpy can identify and extract features of the model output. If we give the features argument to UncertaintyQuantification, Uncertainpy will perform uncertainty quantification and sensitivity analysis of the given features, in addition to the analysis of the raw output data (if desired).

Three sets of features come predefined with Uncertainpy, SpikingFeatures, EfelFeatures, and NetworkFeatures. Each feature class contains a set of features tailored toward one specific type of neuroscience models. We first explain how to create custom features, before explaining how to use the built-in features.

Features are defined through the Features class:


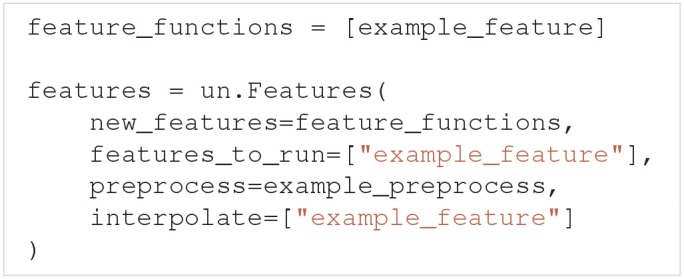


The new_features argument is a list of Python functions that each calculates a specific feature, whereas features_to_run specifies which of the features to perform uncertainty quantification of. If nothing is specified, the uncertainty quantification is by default performed on all features (features_to_run="all"). preprocess is a Python function that performs common calculations for all features. interpolate is a list of features that are irregular. As with models, Uncertainpy automatically interpolates the output of these features to a regular form. Below we first go into detail on the requirements of a feature function, and then the requirements of a preprocess function.

#### 3.4.1. Feature functions

A feature is given as a Python function. The outline of such a feature function is:


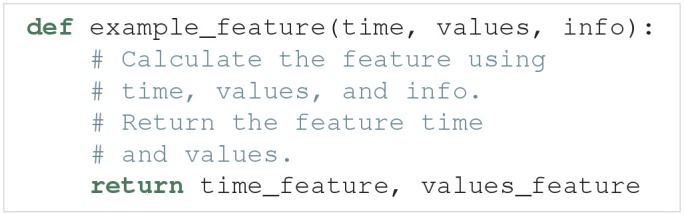


Feature functions have the following requirements:

1. **Input**. The feature function takes the objects returned by the model function as input, except when a preprocess function is used (see below). In those cases, the feature function instead takes the objects returned by the preprocess function as input. preprocess is normally not used.
2. **Feature calculation**. The feature function calculates the value of a feature from the data given in time, values and optional info objects. As previously mentioned, in all built-in features in Uncertainpy, info is a dictionary containing required information as key-value pairs.
3. **Output**. The feature function must return two objects:
  - **Feature time** (time_feature). The time (or equivalent) of the feature. We can return None instead for features where this is not relevant.
  - **Feature values** (values_feature). The result of the feature calculation. As for the model output, the feature result must be regular, or able to be interpolated. If there are no feature result for a specific model evaluation (e.g., if the feature was spike width and there were no spikes), the feature function can return None. The specific feature evaluation is then discarded in the uncertainty calculations.

As with models, we can, as a shortcut, directly give a list of feature functions as the feature argument in UncertaintyQuantification, instead of first having to create a Features instance.

#### 3.4.2. Feature pre-processing

Some of the calculations needed to quantify features may overlap between different features. One example is finding the spike times from a voltage trace. The preprocess function is used to avoid having to perform the same calculations several times. An example outline of a preprocess function is:


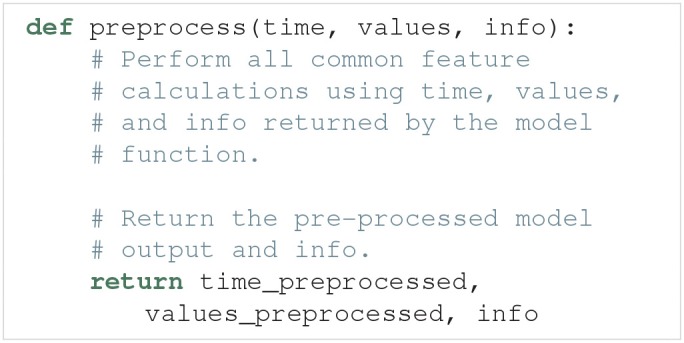


The requirements for a preprocess function are:

1. **Input**. A preprocess function takes the objects returned by the model function as input.
2. **Pre-processing**. The model output (time, values, and additional info objects) are used to perform all pre-process calculations.
3. **Output**. The preprocess function can return any number of objects as output. The returned pre-process objects are used as input arguments to the feature functions, so the two must be compatible.

Figure 2 illustrates how the objects returned by the model function are passed to preprocess, and the returned pre-process objects are used as input arguments in all feature functions. This pre-processing makes feature functions have different required input arguments depending on the feature class they are added to. As mentioned earlier, Uncertainpy comes with three built-in feature classes. These classes all take the new_features argument, so custom features can be added to each set of features. These feature classes all perform a pre-processing and therefore have different requirements for the input arguments of new feature functions. Additionally, certain features require specific keys to be present in the info dictionary. Each class has a reference_feature method that states the requirements for feature functions of that class in its docstring.

#### 3.4.3. Spiking features

Here we introduce the SpikingFeatures class, which contains a set of features relevant for models of single neurons that receive an external stimulus and respond by producing a series of action potentials, also called spikes. Many of these features require the start time and end time of the stimulus, which must be returned as info["stimulus_start"] and info["stimulus_end"] in the model function. info is then used as an additional input argument in the calculation of each feature. A set of spiking features is created by:


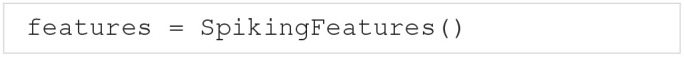


SpikingFeatures implements a preprocess method, which locates spikes in the model output. This preprocess method can be customized; see the documentation on SpikingFeatures.

The features included in SpikingFeatures are briefly defined below. This set of features was taken from the previous work of Druckmann et al. (2007), with the addition of the number of action potentials during the stimulus period. We refer to the original publication for more detailed definitions.

1. nr_spikes – Number of action potentials (during stimulus period).
2. spike_rate – Action-potential firing rate (number of action potentials divided by stimulus duration).
3. time_before_first_spike – Time from stimulus onset to first elicited action potential.
4. accommodation_index – Accommodation index (normalized average difference in length of two consecutive interspike intervals).
5. average_AP_overshoot – Average action-potential peak voltage.
6. average_AHP_depth – Average afterhyperpolarization depth (average minimum voltage between action potentials).
7. average_AP_width – Average action-potential width taken at the midpoint between the onset and peak of the action potential.

The user may want to add custom features to the set of features in SpikingFeatures. The SpikingFeatures.preprocess method changes the input given to the feature functions, and as such each spiking feature function has the following input arguments:

1. The time array returned by the model simulation.
2. A Spikes object (spikes) which contain the spikes found in the model output.
3. An info dictionary with info["stimulus_start"] and info["stimulus_end"] set.

The Spikes object is the pre-processed version of the model output, used as a container for Spike objects. In turn, each Spike object contains information about a single spike. This information includes a brief voltage trace represented by a time and a voltage (V) array that only includes the selected spike. The information in Spikes is used to calculate each feature. As an example, let us create a feature that is the time at which the first spike in the voltage trace ends. Such a feature can be defined as follows:


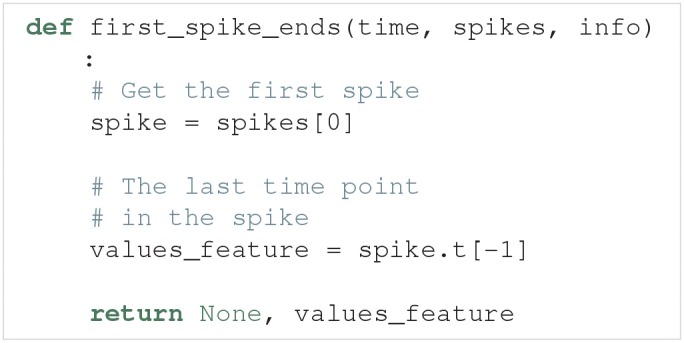


This feature may now be used as a feature function in the list given to the new_features argument.

From the set of both built-in and user-defined features, we may select subsets of features that we want to use in the analysis of a model. Let us say we are interested in how the model performs in terms of the three features: nr_spikes, average_AHP_depth and first_spike_ends. A spiking features object that calculates these features is created by:


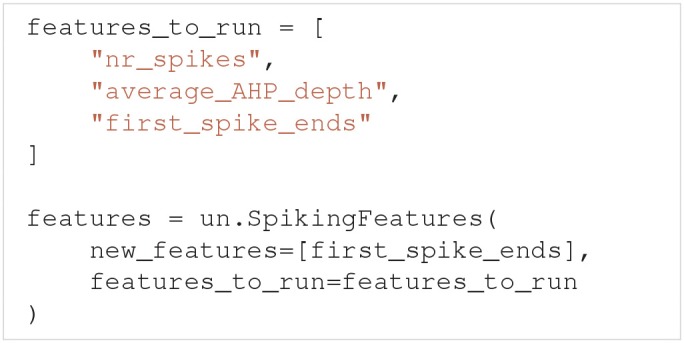


#### 3.4.4. eFEL features

A more extensive set of features for single neuron voltage traces is found in the Electrophys Feature Extraction Library (eFEL) (Blue Brain Project, 2015). A set of eFEL spiking features is created by:


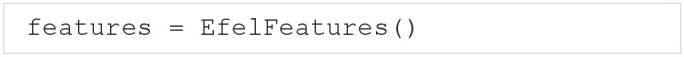


Uncertainpy has all features in the eFEL library in the EfelFeatures class. At the time of writing, eFEL contains 160 different features. Due to the high number of features, we do not list them here, but refer to the eFEL documentation^5^ for detailed definitions, or the Uncertainpy documentation for a list of the features. EfelFeatures is used in the same way as SpikingFeatures.

#### 3.4.5. Network features

The last set of features implemented in Uncertainpy is found in the NetworkFeatures class:


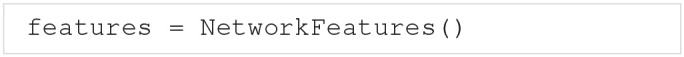


This class contains a set of features relevant for the output of neural network models. These features are calculated using the *Elephant* Python package (NeuralEnsemble, 2017). The implemented features are:

1. average_firing_rate – Average firing rate (for a single recorded neuron).
2. instantaneous_rate – Instantaneous firing rate (averaged over all recorded neurons within a small time window).
3. average_isi – Average interspike interval (averaged over all recorded neurons).
4. cv – Coefficient of variation of the interspike interval (for a single recorded neuron).
5. average_cv – Average coefficient of variation of the interspike interval (averaged over all recorded neurons).
6. local_variation – Local variation (variability of interspike intervals for a single recorded neuron).
7. average_local_variation – Average local variation (variability of interspike intervals averaged over all recorded neurons).
8. fanofactor – Fanofactor (variability of spike trains).
9. victor_purpura_dist – Victor-Purpura distance (spike train dissimilarity between two recorded neurons).
10. van_rossum_dist – Van Rossum distance (spike train dissimilarity between two recorded neurons).
11. binned_isi – Histogram of the interspike intervals (for all recorded neurons).
12. corrcoef – Pairwise Pearson's correlation coefficients (between the binned spike trains of two recorded neurons).
13. covariance – Covariance (between the binned spike trains of two recorded neurons).

A few of these network features can be customized; see the documentation on NetworkFeatures for a further explanation.

The use of NetworkFeatures in Uncertainpy follows the same logic as the use of the other feature classes, and custom features can easily be included. As with SpikingFeatures, NetworkFeatures implements a preprocess method. This preprocess returns the following objects:

1. End time of the simulation (end_time).
2. A list of NEO (Garcia et al., 2014) spike trains (spiketrains).

Each feature function added to NetworkFeatures therefore requires these objects as input arguments. Note that the info object is not used.

### 3.5. Uncertainty calculations in uncertainpy

In this section, we describe how Uncertainpy performs the uncertainty calculations, as well as which options the user has to customize the calculations. Moreover, a detailed insight into this is not required to use Uncertainpy, as in most cases the default settings work fine. In addition to the customization options shown below, Uncertainpy has support for implementing entirely custom uncertainty-quantification and sensitivity-analysis methods. This is only recommended for expert users, as knowledge of both Uncertainpy and uncertainty quantification is needed. We do not go into detail here but refer to the Uncertainpy documentation for more information.

#### 3.5.1. Quasi-Monte Carlo method

To use the quasi-Monte Carlo method, we call quantify with method="mc", and the optional argument nr_mc_samples:


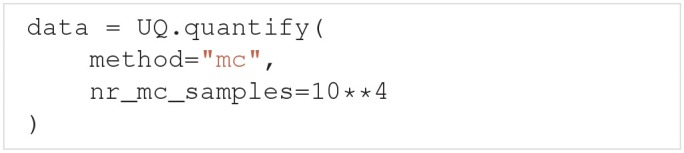


The quasi-Monte Carlo method quasi-randomly draws *N*_*s*_ = *N*(*d* + 2)/2 parameter samples, where *N* = nr_mc_samples, and *d* is the number of uncertain parameters. This is the number of samples required by Saltelli's method to calculate the Sobol indices. By default nr_mc_samples=10000. These samples are drawn from a multivariate independent uniform distribution using Saltelli's sampling scheme, implemented in the SALib library (Saltelli et al., 2010; Herman and Usher, 2017). We use the Rosenblatt transformation to transform the samples from this uniform distribution to the parameter distribution given by the user. This transformation enables us to use Saltelli's sampling scheme for any parameter distribution.

The model is evaluated for each of these parameter samples, and features are calculated from each model evaluation (when applicable). To speed up the calculations, Uncertainpy uses the *multiprocess* Python package (McKerns et al., 2012) to perform this step in parallel. When model and feature calculations are done, Uncertainpy calculates the mean, variance, and 5th and 95th percentile (which gives the 90% prediction interval) for the model and each feature. This is done using a subset with *N* number of samples of the total set. We are unable to use the full set since not all samples are independent in Saltelli's sampling scheme. The Sobol indices are calculated using Saltelli's method and the complete set of samples. We use a modified version of the method in the SALib library, which is able to handle model evaluations with any number of dimensions.

Saltelli's method requires all model and feature evaluations to return a valid result. When this is not the case we use the workaround^6^ suggested by Herman and Usher (2017), and replace invalid model and feature evaluations with the mean of that model or feature. This workaround introduces an error depending on the number of missing evaluations but enables us to still calculate the Sobol indices. If there are invalid model or feature evaluations, Uncertainpy gives a warning which includes the number of invalid evaluations.

#### 3.5.2. Polynomial chaos expansions

To use polynomial chaos expansions we use quantify with the argument method="pc", which takes a set of optional arguments (the specified values are the default):


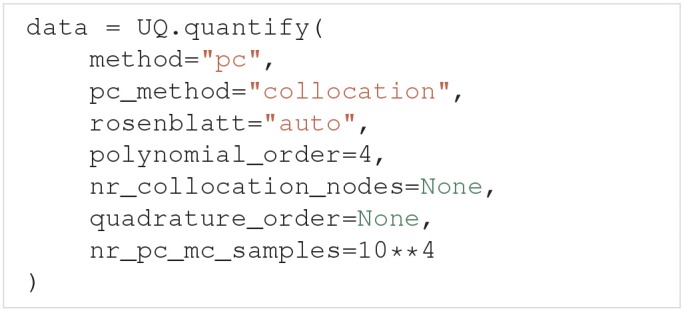


As previously mentioned, Uncertainpy allows the user to select between point collocation (pc_method="collocation") and pseudo-spectral projections (pc_method="spectral"). The goal of both these methods is to create separate polynomial chaos expansions Û_model/feature_ for the model and each feature. The first step of both methods is the same: Uncertainpy starts by creating the orthogonal polynomial **ϕ**_*n*_ using ρ_***Q***_ and the three-term recurrence relation if available, otherwise the discretized Stieltjes method (Stieltjes, 1884) is used. By default, Uncertainpy uses a fourth order polynomial expansion, as recommended by Eck et al. (2016). The polynomial order *p* can be changed with the polynomial_order argument. The polynomial **ϕ**_*n*_ is the same for the model and all features, since they have the same uncertain input parameters, and therefore the same ρ_***Q***_. Only the polynomial coefficients *c*_*n*_ differ between the model and each feature.

The two polynomial chaos methods differ in terms of how they calculate *c*_*n*_. For point collocation Uncertainpy uses *N*_*s*_ = 2(*N*_*p*_ + 1) collocation nodes, as recommended by Hosder et al. (2007), where *N*_*p*_ is the number of polynomial chaos expansion factors. The number of collocation nodes can be customized with nr_collocation_nodes (*N*_*s*_), but the new number of nodes must be chosen carefully. The collocation nodes are sampled from ρ_***Q***_ using Hammersley sampling (Hammersley, 1960), also as recommended by Hosder et al. (2007). The model and features are calculated for each of the collocation nodes. As with the quasi-Monte Carlo method, this step is performed in parallel. The polynomial coefficients *c*_*n*_ are calculated using the model and feature results, and Tikhonov regularization (Rifkin and Lippert, 2007).

For the pseudo-spectral projection, Uncertainpy chooses nodes and weights using a quadrature scheme, instead of choosing nodes from ρ_***Q***_. The quadrature scheme used is Leja quadrature with a Smolyak sparse grid (Smolyak, 1963; Narayan and Jakeman, 2014). The Leja quadrature is by default of order two greater than the polynomial order, but this can be changed with quadrature_order. The model and features are calculated for each of the quadrature nodes. As before, this step is performed in parallel. The polynomial coefficients *c*_*n*_ are then calculated from the quadrature nodes, weights, and model and feature results.

When Uncertainpy has derived Û for the model and features, it uses Û to compute the mean, variance, first and total-order Sobol indices, as well as the average first and total-order Sobol indices. Finally, Uncertainpy uses Û as a surrogate model and employs the quasi-Monte Carlo method with Hammersley sampling and nr_pc_mc_samples=10**4 samples to find the 5th and 95th percentiles.

If the model parameters have a dependent joint multivariate distribution, the Rosenblatt transformation is by default automatically used. This can be changed by setting rosenblatt=True to always use the Rosenblatt transform, or rosenblatt=False to never use the Rosenblatt transformation. Note that the latter gives an error if you have dependent parameters. To perform this transformation Uncertainpy chooses a multivariate independent normal distribution ρ_***R***_, which is used instead of ρ_***Q***_ to perform the polynomial chaos expansions. Both the point-collocation method and the pseudo-spectral method are performed as described above. The only difference is that we use ρ_***R***_ instead of ρ_***Q***_, and use the Rosenblatt transformation to transform the selected nodes from ***R*** to ***Q***, before they are used in the model evaluation.

### 3.6. Data format

All results from the uncertainty quantification and sensitivity analysis are returned as a Data object, as well as being stored in UncertaintyQuantification.data. The Data class works similarly to a Python dictionary. The names of the model and features are the keys, while the values are DataFeature objects that store each statistical metric in Table 1 as attributes. Results can be saved and loaded through Data.save and Data.load, and are saved either as HDF5 files (Collette, 2013) or Exdir structures (Dragly et al., 2018). HDF5 files are used by default.

**Table 1:** Calculated values and statistical metrics, for the model and each feature stored in the Data class.

**Calculated statistical metric**	**Symbol**	**Variable**
Model and feature evaluations	*U*	evaluations
Model and feature times	*t*	time
Mean	𝔼	mean
Variance	*V*	variance
5th percentile	*P*_5_	percentile_5
95th percentile	*P*_95_	percentile_95
First-order Sobol indices	*S*	sobol_first
Total-order Sobol indices	*S*_*T*_	sobol_total
Average of the first-order Sobol indices	$\bar{S}$	sobol_first_average
Average of the total-order Sobol indices	${\bar{S}}_{T}$	sobol_total_average

An example: If we have performed an uncertainty quantification of a spiking neuron model with the number of spikes as one of the features, we can load the results and get the variance of the number of spikes by:


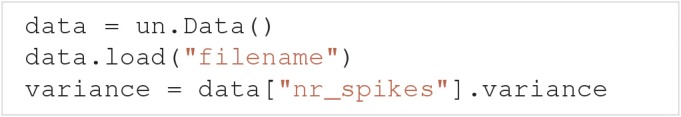


### 3.7. Visualization

Uncertainpy plots the results for all zero and one-dimensional statistical metrics, and some of the two-dimensional statistical metrics. An example of a zero-dimensional statistical metric is the mean of the average interspike interval of a neural network (**Figure 8**). An example of a one-dimensional statistical metric is the mean of the membrane potential over time for a multi-compartmental neuron (**Figure 4**). Lastly, an example of a two-dimensional statistical metric is the mean of the pairwise Pearson's correlation coefficient of a neural network (**Figure 9**). These visualizations are intended as a quick way to get an overview of the results, and not to create publication-ready plots. Custom plots of the data can easily be created by retrieving the results from the Data class.

### 3.8. Technical aspects

Uncertainpy is open-source and found at https://github.com/simetenn/uncertainpy. Uncertainpy can easily be installed using pip:


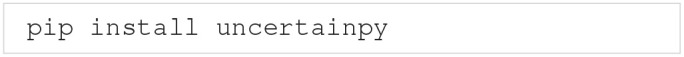


or from source by cloning the Github repository:


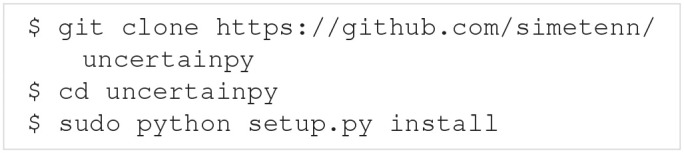


Uncertainpy comes with an extensive test suite that can be run with the test.py script. For information on how to use test.py, run:


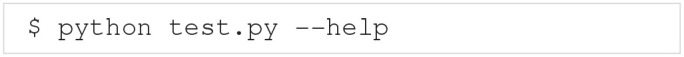


## 4. Example applications

In the current section, we demonstrate how to use Uncertainpy by applying it to four different case studies: (i) a simple model for the temperature of a cooling coffee cup implemented in Python, (ii) the original Hodgkin-Huxley model implemented in Python, (iii) a multi-compartmental model of a thalamic interneuron implemented in NEURON, and (iv) a sparsely connected recurrent network model implemented in NEST. The codes for all four case studies are available in uncertainpy/examples/, which generates all results shown in this paper. All the case studies can be run on a regular workstation computer. Uncertainpy does not create publication-ready figures, so custom plots have been created for the case studies. The code for creating all figures in this paper is found in a Jupyter Notebook in uncertainpy/examples/paper_figures/.

For simplicity, uniform distributions were assumed for all parameter uncertainties in the example studies. Further, the results for the case studies are calculated using point collocation. For the examples shown we used the default polynomial order of *p* = 4, but also confirmed that the results converged by increasing the polynomial order to *p* = 5, which gave similar results (results not shown).

The case studies were run in a Docker^7^ container with Python 3, created from the Dockerfile uncertainpy/.docker/Dockerfile_uncertainpy3. A similar Dockerfile is available for Python 2. The used version of Uncertainpy is 1.0.1, commit b7b3fa0, and Zenodo^8^ DOI 10.5281/zenodo.1300336. We also used NEST 2.14.0, NEURON 7.5, and Chaospy commit 05fea24. A requirements file that specifies the version of all used Python packages is located in uncertainpy/examples/paper_figures/.

### 4.1. Cooling coffee cup

To give a simple, first demonstration of Uncertainpy, we perform an uncertainty quantification and sensitivity analysis of a hot cup of coffee that follows Newton's law of cooling. We start with a model that has independent uncertain parameters, before we modify the model to have dependent parameters to show an example requiring the Rosenblatt transformation.

#### 4.1.1. Cooling coffee cup with independent parameters

The temperature *T* of the cooling coffee cup is given by:

(18) $$\begin{array}{l} \frac{dT\left(t\right)}{dt}=-\kappa \left(T\left(t\right)-T_{env}\right), \end{array}$$

where *T*_*env*_ is the temperature of the environment in units of °C. κ is a cooling constant in units of 1/min that is characteristic of the system and describes how fast the coffee cup radiates heat to the environment. We set the initial temperature to a fixed value, *T*_0_ = 95°C, and assume that κ and *T*_*env*_ are uncertain parameters characterized by the uniform probability distributions:

(19) $$\begin{array}{l} \rho_{\kappa }=\text{Uniform}\left(0.025,0.075\right), \end{array}$$

(20) $$\begin{array}{l} \rho_{T_{env}}=\text{Uniform}\left(15,25\right). \end{array}$$

The following code is available in uncertainpy/examples/coffee_cup/. We start by importing the packages required to perform the uncertainty quantification:


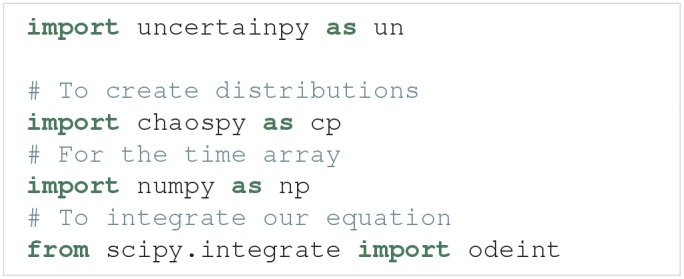


Next, we create the cooling coffee-cup model. To do this we define a Python function (coffee_cup) that takes the uncertain parameters kappa and T_env as input arguments, solves Equation (18) by integration using scipy.integrate.odeint over 200 min, and returns the resulting time and temperature arrays.


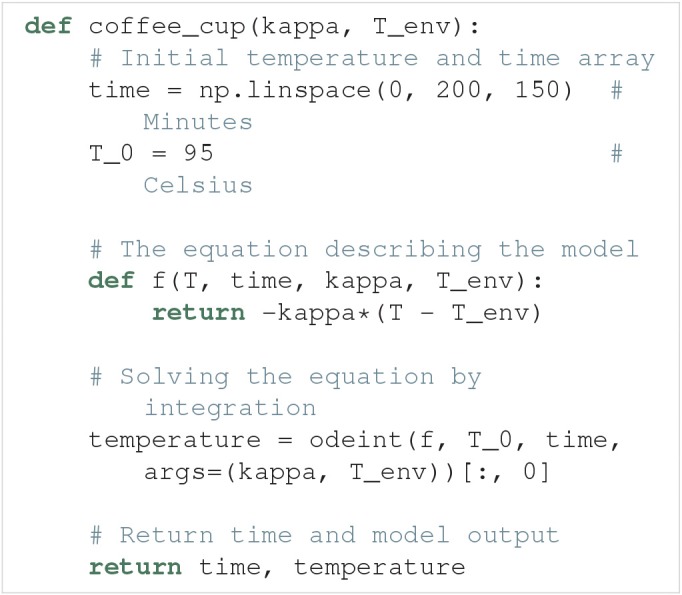


We now use coffee_cup to create a Model object, and add labels:


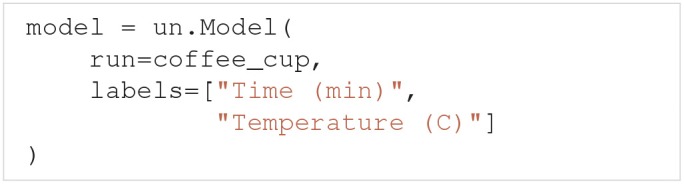


As previously mentioned, it is possible to use coffee_cup directly as the model argument in the UncertaintyQuantification class, however we would then be unable to specify the labels.

In the next step, we use Chaospy to assign distributions to the uncertain parameters κ and *T*_*env*_, and use these distributions to create a parameter dictionary:


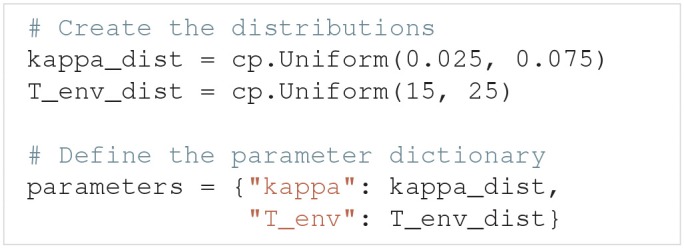


We can now set up the UncertaintyQuantification:


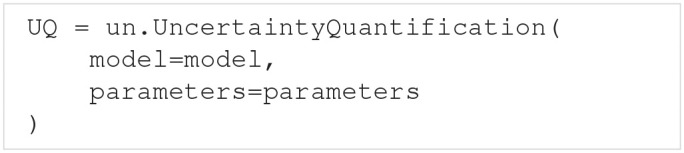


**Figure 3:**
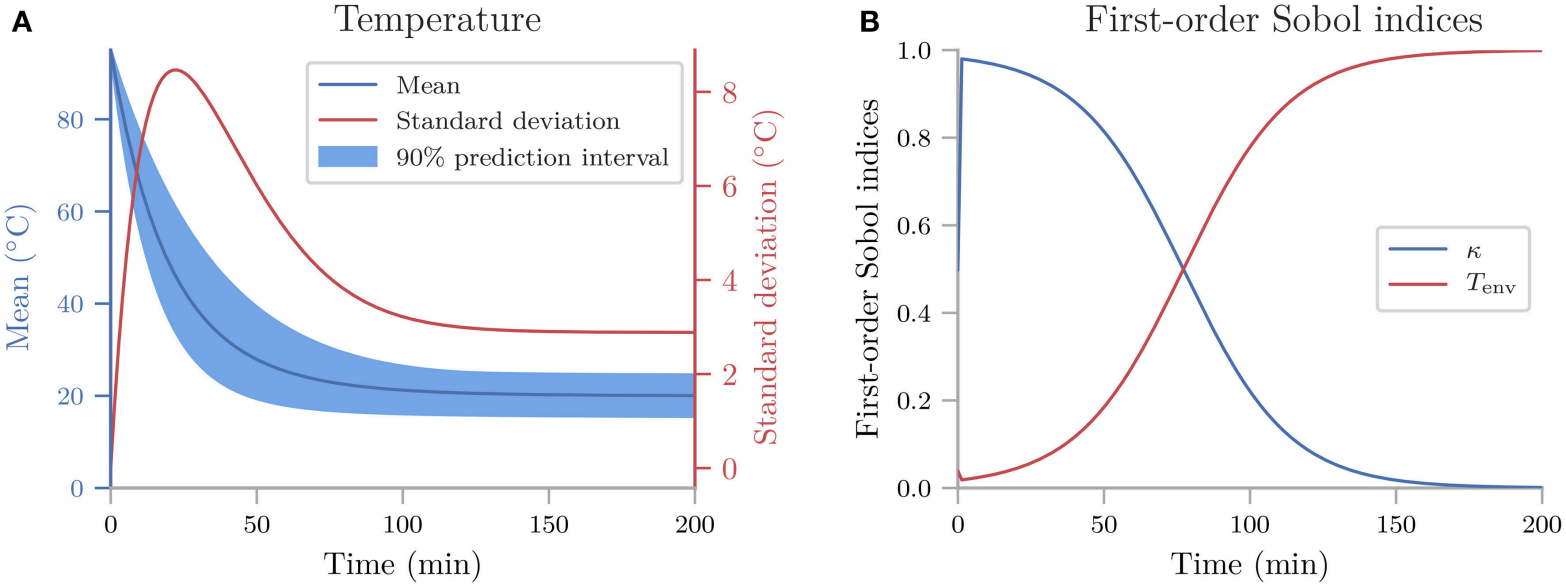
The uncertainty quantification and sensitivity analysis of the cooling coffee-cup model. **(A)** The mean, standard deviation (square root of the variance) and 90% prediction interval of the temperature of the cooling coffee cup. **(B)** First-order Sobol indices of the cooling coffee-cup model.

With that, we are ready to calculate the uncertainty and sensitivity of the model. We use polynomial chaos expansions with point collocation, the default options of quantify, and set the seed for the random number generator to allow for precise reproduction of the results:


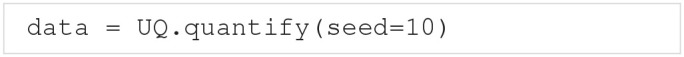


quantify calculates all statistical metrics discussed in sections 2.2 and 2.3, but here we only show the mean, standard deviation (square root of the variance), and 90% prediction interval (Figure 3A), and the first-order Sobol indices (Figure 3B). The reason we plot the standard deviation instead of the variance is to make it easier to compare it to the mean. As the mean (blue line) in Figure 3A shows, the cooling gives rise to an exponential decay in the temperature, toward the temperature of the environment *T*_env_. From the sensitivity analysis (Figure 3B) we see that *T* is most sensitive to κ early in the simulation, and to *T*_env_ toward the end of the simulation. This is as expected since κ determines the rate of the cooling, while *T*_env_ determines the final temperature. After about 150 min, the cooling is essentially completed, and the uncertainty in *T* exclusively reflects the uncertainty of *T*_env_.

#### 4.1.2. Cooling coffee cup with statistically dependent parameters

Uncertainpy can also perform uncertainty quantification and sensitivity analysis using polynomial chaos expansions on models with statistically dependent parameters. Here we use the cooling coffee-cup model to construct such an example. Let us parameterize the coffee cup differently:

(21) $$\begin{array}{l} \frac{dT\left(t\right)}{dt}=-\alpha \hat{\kappa }\left(T\left(t\right)-T_{env}\right). \end{array}$$

In order for the model to describe the same cooling process as before, the new variables α and $\hat{\kappa }$ should be dependent, so that $\alpha \hat{\kappa }=\kappa$. We can achieve this by demanding that $\rho_{\hat{\kappa }}=\rho_{\kappa }/\rho_{\alpha }$ (note that ρ_α_ should not include 0) and otherwise define the problem following the same procedure as in the original case study. Since this gives us a dependent distribution, Uncertainpy automatically uses the Rosenblatt transformation.

In this case, the distribution we assign to α does not affect the end result, as the distribution for $\hat{\kappa }$ will be scaled accordingly. Using the Rosenblatt transformation, an uncertainty quantification and sensitivity analysis of the dependent coffee-cup model therefore return the same results as seen in Figure 3, where the role of the original κ is taken over by $\hat{\kappa }$, while the sensitivity to the additional parameter α becomes strictly zero (we do not show the results here, but see the example in uncertainpy/examples/coffee_cup_dependent/).

### 4.2. Hodgkin-Huxley model

From here on, we focus on case studies more relevant for neuroscience, starting with the original Hodgkin-Huxley model (Hodgkin and Huxley, 1952). An uncertainty analysis of this model has been performed previously (Torres Valderrama et al., 2015), and we here repeat a part of that study using Uncertainpy.

The original version of the Hodgkin-Huxley model has eleven parameters with the numerical values listed in Table 2. As in the previous study, we assume each of these parameters has a uniform distribution in the range ±10% around their original value. We use uncertainty quantification and sensitivity analysis to explore how these parameter uncertainties affect the model output, i.e., the action potential response of the neural membrane potential to an external current injection.

**Table 2:** Parameters in the original Hodgkin-Huxley model.

**Parameter**	**Value**	**Unit**	**Meaning**
*V*_0_	−10	mV	Initial voltage
*C*_m_	1	μF/cm^2^	Membrane capacitance
ḡ_Na_	120	mS/cm^2^	Maximum sodium (Na) conductance
ḡ_K_	36	mS/cm^2^	Maximum potassium (K) conductance
ḡ_L_	0.3	mS/cm^2^	Maximum leak current conductance
*E*_Na_	112	mV	Sodium equilibrium potential
*E*_K_	−12	mV	Potassium equilibrium potential
*E*_L_	10.613	mV	Leak current equilibrium potential
*n*_0_	0.0011		Initial potassium activation gating variable
*m*_0_	0.0003		Initial sodium activation gating variable
*h*_0_	0.9998		Initial sodium inactivation gating variable

As in the cooling coffee-cup example, we implement the Hodgkin-Huxley model as a Python function and use polynomial chaos expansions with point collocation to calculate the uncertainty and sensitivity of the model (the code for this case study is found in uncertainpy/examples/valderrama/).

**Figure 4:**
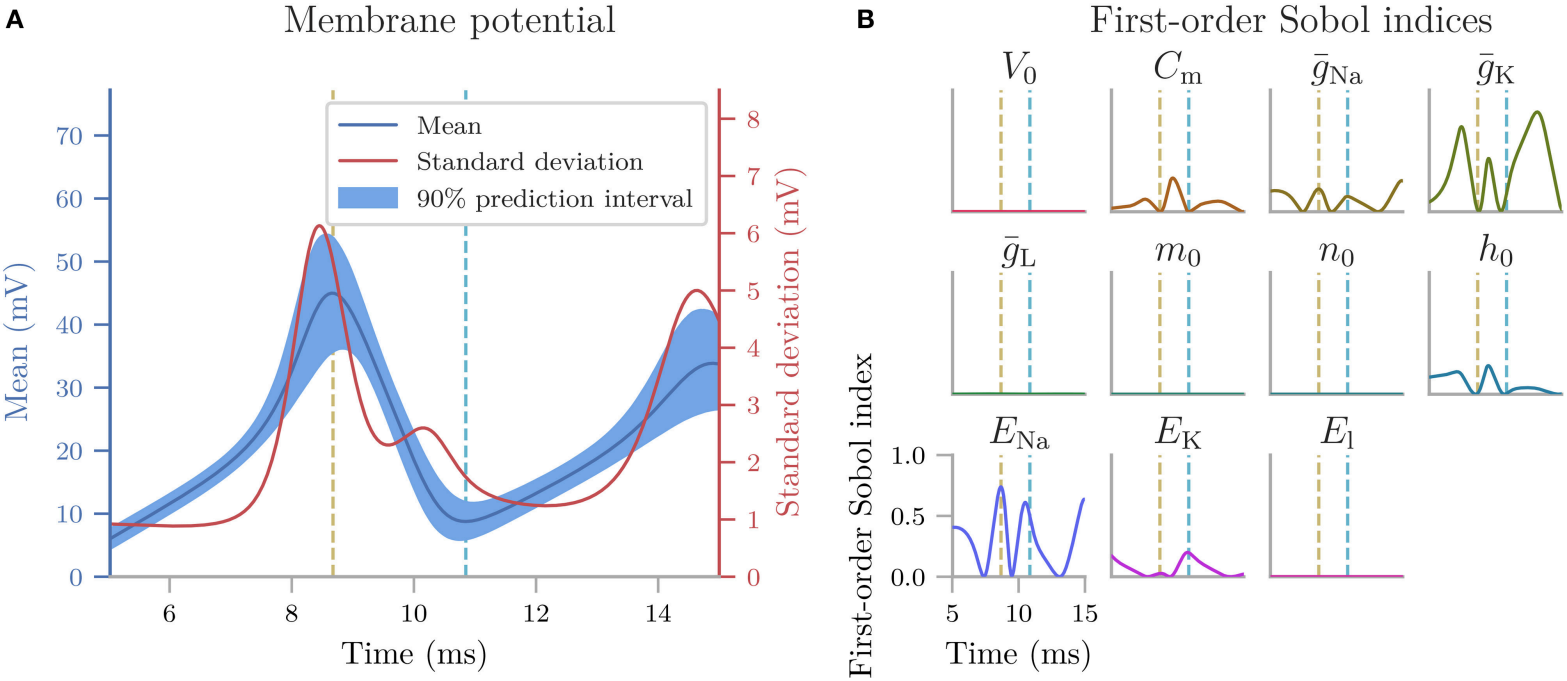
The uncertainty quantification and sensitivity analysis of the Hodgkin-Huxley model, parameterized so it has a resting potential of 0 mV. The model was exposed to a continuous external stimulus of 140 μA/cm^2^ starting at *t* = 0, and we examined the membrane potential in the time window between *t* = 5 and 15 ms. **(A)** Mean, standard deviation and 90% prediction interval for the membrane potential of the Hodgkin-Huxley model. **(B)** First-order Sobol indices of the uncertain parameters in the Hodgkin-Huxley model. The yellow line indicates the peak of the first action potential, while the cyan line indicates the minimum after the first action potential.

The uncertainty quantification of the Hodgkin-Huxley model is shown in Figure 4A, and the sensitivity analysis in Figure 4B. As we were not able to extract all implementation details in Torres Valderrama et al. (2015), our analysis is likely not an exact replica of the previous study, but the results obtained are quantitatively similar. Although the action potential is robust (within the selected parameter ranges), the onset and amplitude of the action potential vary between simulations. The variance (standard deviation) in the membrane potential is largest during the upstroke and peak of the action potential (Figure 4A), which occur in the time interval between *t* = 8 and 9 ms. This occurs mainly due to the difference in action potential timing.

The sensitivity analysis reveals that the variance in the membrane potential mainly is due to the uncertainty in two parameters: the maximum conductance of the K^+^ channel, ḡ_K_, and the Na^+^ reversal potential, *E*_Na_ (Figure 4B). The low sensitivity to the remaining parameters means that most of the variability of the Hodgkin-Huxley model would be maintained if these remaining parameters were kept fixed. This result tells us that if we want to reduce the uncertainty in the model predictions, experiments should focus on measuring ḡ_K_ and *E*_Na_ more precisely, while crude estimates of the remaining parameters will suffice. Of course, this conclusion only holds for the conditions considered in the current simulation, where the neuron is exposed to positive current injection starting at *t* = 0. If the neuron received no input, the membrane potential would show a much higher sensitivity to the leak current (*E*_L_ and ḡ_L_) which are important for determining the resting potential of the neuron.

A sensitivity analysis such as that in Figure 4B may serve to give an insight into how different mechanisms are responsible for different aspects of the neuronal response. Some of the findings confirm what we would expect from a general knowledge of action potential firing (see figure 3.12 in Sterratt et al., 2011 for an overview). For example, it is not surprising that the action potential peak potential is most sensitive to the Na^+^ reversal potential (*E*_Na_), since this parameter is known to closely determine the peak value. Nor is it surprising that ḡ_K_ is the most important parameter during the downstroke of the action potential, since the essential role of the K^+^ channel is to repolarize the neuron.

Other parts of the analysis reveal some less intuitive relationships. For example, Figure 4B shows that the membrane potential during the upstroke of the action potential is most sensitive to ḡ_K_. This may be surprising given that the Na^+^ channel (parameterized by ḡ_Na_ and *E*_Na_) is responsible for depolarizing the neuron. This indicates that the all-or-nothing response of the Na^+^ channel activation is rather robust, and that variance during the upstroke predominantly is due to the effects of the K^+^ channel on the timing of the action-potential onset. Another unexpected observation is that *E*_Na_ has a high sensitivity within a time window after the peak of the action potential. This indicates that the Na^+^ channel is not fully closed, and is involved in determining the potential at which the neuron lingers within this time window.

Another aspect of modeling where sensitivity analysis can be useful, is in exploring the dependence on initial conditions. When analyzing complex models, it is common to discard the initial part of the simulation from the analysis, i.e., one lets the model run for a time *T* before one starts to analyze its dynamics. The rationale behind this is that the model over time loses its dependence on (arbitrarily set) initial conditions of its dynamic variables, and reaches its inherent steady-state dynamics. In the example studied here, only the response for *T* > 5 ms is analyzed. Figure 4B shows that the Hodgkin-Huxley model then has a negligible sensitivity to the initial membrane potential (*V*_0_) and initial activation states of the Na^+^ channel (*m*_0_) and K^+^ channel (*n*_0_), but maintains a sensitivity to the initial Na^+^ inactivation state (*h*_0_) through most of the simulation. Such a dependence on the initial condition of a state variable is typically unwanted and indicates that the model should have had more time to settle in before its response was analyzed.

### 4.3. Multi-compartmental model of a thalamic interneuron

In the next case study, we illustrate how Uncertainpy can be used on models implemented in NEURON (Hines and Carnevale, 1997). For this study, we select a previously published model of an interneuron in the dorsal lateral geniculate nucleus (dLGN) of the thalamus (Halnes et al., 2011). Since the model is implemented in NEURON, the original model can be used directly with Uncertainpy by using the NeuronModel class. The code for this case study is found in uncertainpy/examples/interneuron/.

In the original modeling study, seven active ion channels were tuned (by trial and error) to capture the responses of thalamic interneurons to different current injections (Halnes et al., 2011). Here, we consider one of the stimulus conditions used in the original study, and examine how sensitive the interneuron response is to uncertain ion-channel conductances. The conductances in the original model are listed in Table 3, and we assume they have uniform distributions in the interval ±10% around their original values.

**Table 3:** Uncertain parameters in the thalamic interneuron model.

**Parameter**	**Value**	**Unit**	**Variable**	**Meaning**
*g*_Na_	0.09	S/cm^2^	gna	Max Na^+^-conductance in soma
*g*_Kdr_	0.37	S/cm^2^	gkdr	Max direct-rectifying K^+^-conductance in soma
*g*_CaT_	1.17 × 10^−5^	S/cm^2^	gcat	Max T-type Ca^2+^-conductance in soma
*g*_CaL_	9 × 10^−4^	S/cm^2^	gcal	Max L-type Ca^2+^-conductance in soma
*g*_h_	1.1 × 10^−4^	S/cm^2^	ghbar	Max conductance of a non-specific
				hyperpolarization activated cation channel in soma
*g*_AHP_	6.4 × 10^−5^	S/cm^2^	gahp	Max afterhyperpolarizing K^+^-conductance in soma
*g*_CAN_	2 × 10^−8^	S/cm^2^	gcanbar	Max conductance of a Ca^2+^-activated
				non-specific cation channel in soma

*For simplicity, we limited the analysis to only explore sensitivity to ion channel conductances, although the original model had some additional free parameters*.

**Figure 5:**
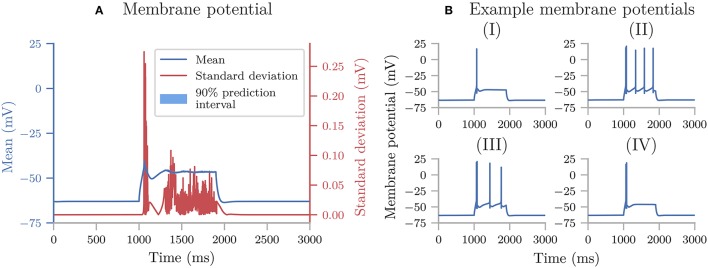
Uncertainty quantification of the interneuron model. **(A)** The mean, standard deviation, and 90% prediction interval for the membrane potential of the interneuron model. **(B)** Four selected model outputs for different sets of parameters. The interneuron received a somatic current injection between 1, 000 ms < *t* < 1, 900 ms, with a stimulus strength of 55 pA.

The uncertainty quantification of the membrane potential in the soma of the interneuron is seen in Figure 5A. The variance (or standard deviation) indicates that the neuronal response varies strongly between the different parameterizations. To illustrate the variety of response characteristics hiding in the statistics in Figure 5A, four selected example simulations are shown in Figure 5B, all obtained by drawing the uncertain parameters from intervals ±10% around their original values. In line with the discussion in section 2.7, the qualitative differences between the responses indicate that a feature-based analysis is more informative than a point-to-point comparison of the voltage traces.

Since we examine a spiking neuron model, we want to use the features in the SpikingFeatures class for the feature-based analysis. SpikingFeatures needs to know the start and end times of the stimulus to be able to calculate certain features. When we initialize NeuronModel we therefore specify the stimulus_start (set to 1,000 ms) and stimulus_end (set to 1,900 ms) arguments. Additionally, the interneuron model uses adaptive time steps, meaning we have to use interpolate=True (which is the default option of NeuronModel). We also specify the path to the folder where the neuron model is stored (for this example, it is path="interneuron_modelDB/"). As before, we use polynomial chaos expansions with point collocation to compute the statistical metrics for the model output and all features.

**Figure 6:**
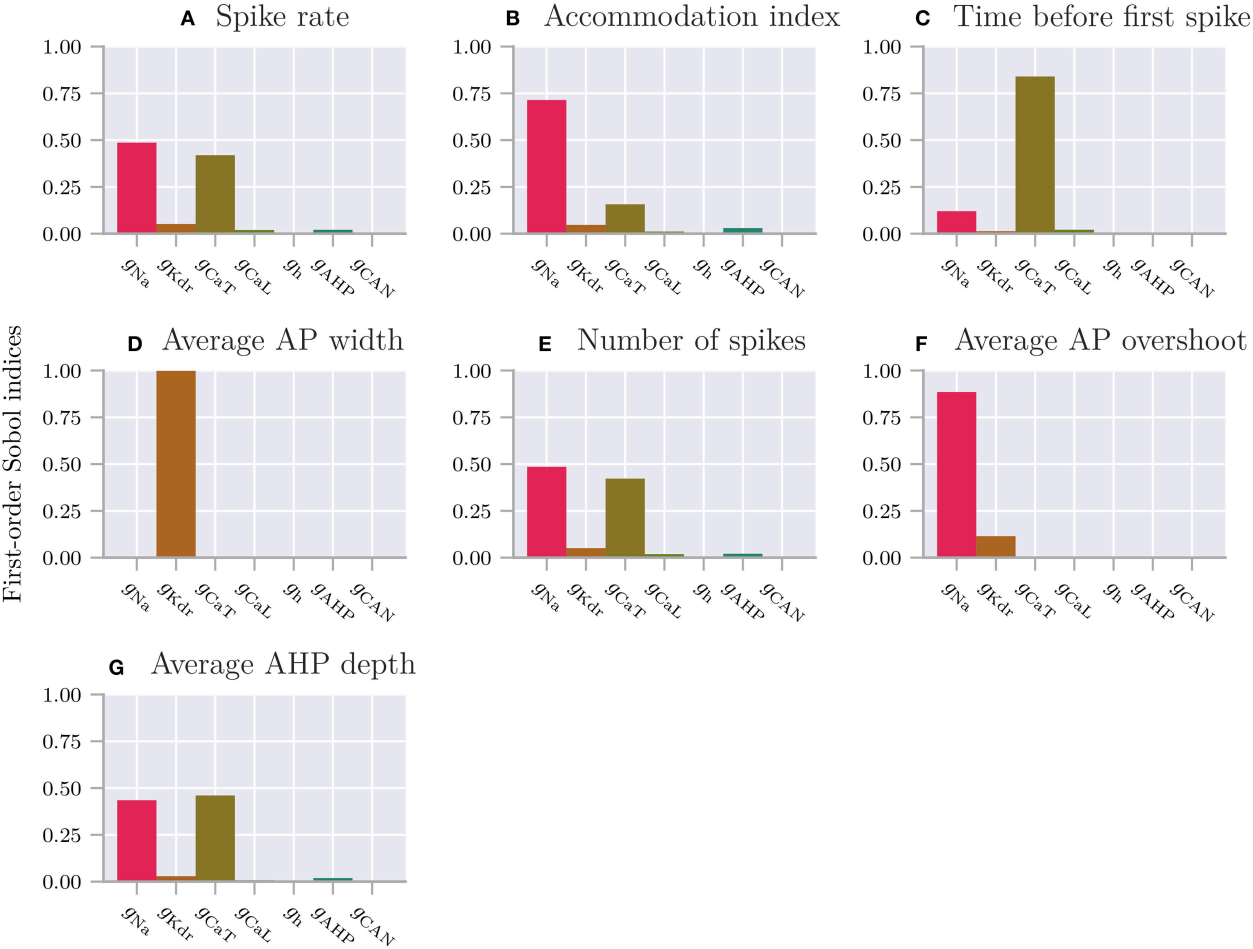
The sensitivity for features of the interneuron model. First-order Sobol indices for features of the thalamic interneuron model. **(A)** Spike rate, that is, number of action potentials divided by stimulus duration. **(B)** Accommodation index, that is, the normalized average difference in length of two consecutive interspike intervals. **(C)** Time before first spike, that is, the time from stimulus onset to first elicited action potential. **(D)** Average AP width is the average action potential width taken at midpoint between the onset and peak of the action potential. **(E)** Number of spikes, that is, the number of action potentials during stimulus period. **(F)** Average AP overshoot is the average action-potential peak voltage. **(G)** Average AHP depth, that is, the average minimum voltage between action potentials.

Figure 6 shows the sensitivity of the features in SpikingFeatures to the various ion-channel conductances (see section 3.4.3 for definitions of the features). For illustrative purposes, only the first-order Sobol indices are shown (although Uncertainpy by default calculates all statistical metrics from sections 2.2 and 2.3).

A feature-based sensitivity analysis such as in Figure 6 gives valuable insight into the role of various biological mechanisms in determining the firing properties of a neuron. Some of the results confirm what we would expect from a general knowledge of neurodynamics. For example, it is not surprising that the spike rate (A), the number of action potentials elicited throughout the simulation (E), and the action-potential amplitude (F) are most sensitive to the Na^+^ channel conductance *g*_Na_, given the well-established role of the Na^+^ channel in action-potential generation. Likewise, given the role of the K^+^ channel in repolarizing the neuron after an action potential, it is not surprising that the action-potential width (D) is predominantly sensitive to *g*_Kdr_.

The third most important parameter identified in this sensitivity analysis is the T-type Ca^2+^ conductance (*g*_CaT_), known to be important for burst firing in thalamic interneurons (Zhu et al., 1999; Halnes et al., 2011; Allken et al., 2014). T-type Ca^2+^ channels are typically activated when the membrane potential makes a sudden step from low to high values, such as at the stimulus onset. Upon activation, T-type Ca^2+^ channels then evoke Ca^2+^ spikes which may act to boost the initial response of a neuron to an external stimulus. This explains why the timing of the first spike (C) has such a high sensitivity to *g*_CaT_. Bursts are typically more pronounced under other stimulus conditions than the one used in the current simulations, but in some cases, the Ca^2+^ spike was large enough to evoke small, initial bursts of action potentials (see example simulations in Figure 5B, II–IV, where the initial responses are small bursts of two action potentials). The additional action potentials in neurons that elicit bursts serve to explain why the spike rate (A) and total number of action potentials (G) also are highly sensitive to *g*_CaT_.

A perhaps less expected result is that the depth of the afterhyperpolarization (G) (voltage dip following an action potential) has such a low sensitivity to the two K^+^ channels (*g*_Kdr_ and *g*_AHP_) in the model, as these are the channels that have a direct effect on the hyperpolarization of the neuron. As for many of the features in Figure 6, there are complex interactions between several mechanisms and the limited analysis considered here can only hint at the possible underlying relationships. Part of the explanation may be that the afterhyperpolarization current (*g*_AHP_) is Ca^2+^ activated, and is more limited by the availability of Ca^2+^ than by its own maximum conductance. This could serve to explain the high sensitivity to the Ca^2+^ channel *g*_CaT_. Furthermore, the high sensitivity to *g*_Na_ implies that the Na^+^ channel also is open during the down-stroke of the action potential, and counteracts the hyperpolarizing K^+^ currents.

As Figure 6 indicates, the variances of the SpikingFeatures are predominantly explained by the three model parameters *g*_Na_, *g*_Kdr_ and *g*_CaT_, with some contributions from *g*_CaL_, *g*_AHP_ and negligible impact from the remaining parameters *g*_h_ and *g*_CAN_. However, one should be cautious about generalizing insights found in an unexhaustive analysis such as the one presented here. Firstly, the presented analysis explores the sensitivity to variations within a ±10% range around the original parameter values, and thus spans a relatively local region of the parameter space. Additionally, this choice of distributions is a rather arbitrary choice and is unlikely to capture the actual uncertainty distributions. In reality, the uncertainty or biological variability, or both, in some of the parameters may have very different distributions, and an analysis that takes this into account could yield different results. Secondly, the above analysis was limited to a single stimulus protocol (a positive current step pulse of moderate magnitude to the soma), and a different stimulus protocol could activate a different set of neural mechanisms. For example, *g*_h_ denotes the conductance of a hyperpolarization-activated cation current, which would need a negative current injection to activate. It is therefore not surprising that our analysis shows zero sensitivity to this parameter.

Thirdly, the SpikingFeatures class contains a limited number of features, and other features (e.g., from the more comprehensive EfelFeatures class) can be sensitive to the parameters that were observed to be of less importance in the current example. We do not here consider additional features, stimulus protocols, or uncertainty distributions in the analysis, as the main purpose of this case study was to demonstrate the use of Uncertainpy on a detailed multi-compartmental model.

### 4.4. Recurrent network of integrate-and-fire neurons

In the last case study, we use Uncertainpy to perform a feature-based analysis of the sparsely connected recurrent network of integrate-and-fire neurons by Brunel (2000). We implement the Brunel network using NEST inside a Python function, and create 10,000 excitatory and 2,500 inhibitory neurons, with properties as specified by Brunel (2000). Each neuron has 1,000 randomly chosen connections to excitatory neurons and 250 randomly chosen connections to inhibitory neurons (a connection probability of ϵ = 0.1). The weight of the excitatory synapses (amplitude of excitatory synaptic current) is *J* = 0.1 mV. We simulate the network for 1,000 ms, record the output from 20 of the excitatory neurons, and start the recording after 100 ms. The code for this case study is found in uncertainpy/examples/brunel/.

Three more parameters are needed to specify the Brunel model: (i) the external input rate (ν_ext_) relative to the threshold rate (ν_thr_) given as η = ν_ext_/ν_thr_, (ii) the relative strength of the inhibitory synapses compared to the excitatory synapses *g*, and (iii) the synaptic delay *D*. Depending on these parameters, the Brunel network may be in several different activity states. For the current case study we limit our analysis to two of these states, the synchronous regular (SR) state, where the neurons are almost completely synchronized, and the asynchronous irregular (AI) state, where the neurons fire mostly independently at low rates.

**Figure 7:**
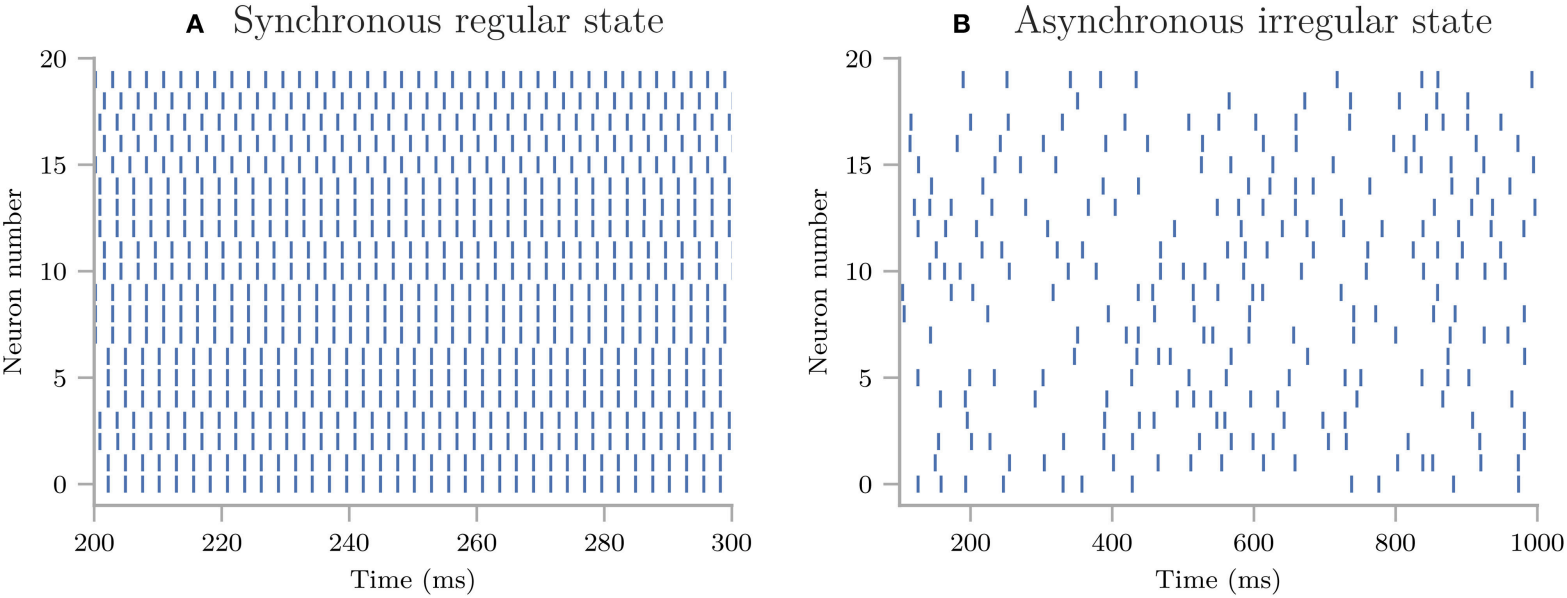
Example model results for the Brunel network. **(A)** The recorded spike train for the Brunel network in the synchronous regular state between 200 and 300 ms of the simulation. **(B)** The recorded spike trains for the Brunel network in the asynchronous irregular state for the entire simulation period. The network has 10, 000 excitatory and 2, 500 inhibitory neurons, with properties as specified by Brunel (2000). Each neuron has 1, 000 randomly chosen connections to excitatory neurons and 250 randomly chosen connections to inhibitory neurons. We simulate the network for 1, 000 ms, record the output from 20 of the excitatory neurons, and start the recording after 100 ms.

We create two sets of model parameters, one for the SR state and one for the AI state. For each set we assume that the uncertainties of the parameters η, *g* and *D* are characterized by uniform probability distributions within the ranges shown in Table 4. The parameter ranges are chosen so that all parameter combinations within the set give model behavior corresponding to one of the states. Two selected model results representative of the network in both states are shown in Figure 7, which illustrate the differences between the two states. Figure 7 shows the recorded spike trains for the Brunel network in the SR state between 200 ms and 300 ms of the simulation. The results in this time window exemplifies network behavior during the entire simulation after spiking has started. Since the firing rate is very high in this state, only results for a limited time window are shown. Figure 7B shows the recorded spike trains for the Brunel network in the AI state for the entire simulation period.

**Table 4:** Parameters in the Brunel network for the asynchronous irregular (AI) and synchronous regular (SR) state.

**Parameter**	**Range SR**	**Range AI**	**Variable**	**Meaning**
η	[1.5, 3.5]	[1.5, 3.5]	eta	External rate relative to threshold rate
*g*	[1, 3]	[5, 8]	g	Relative strength of inhibitory synapses
*D*	[1.5, 3]	[1.5, 3]	delay	Synaptic delay (ms)

*Each parameter has a uniform distribution within the given range*.

We use the features in NetworkFeatures to examine features of the network dynamics. Of the 13 built-in network features in NetworkFeatures, we here only focus on two: the average interspike interval and the pairwise Pearson's correlation coefficient. These features are well suited to highlight the differences between the AI and SR network states, and to investigate how the details of the network dynamics depend on the model parameters within each of the states. We perform an uncertainty quantification and sensitivity analysis of the model and all features for each of the network states using polynomial chaos with point collocation. As for the previous examples we used the default polynomial order of *p* = 4 which was observed to be sufficient to achieve convergence, that is, the results did not change much when increasing *p* beyond 4.

We also explored the alternative situation where the excitatory synaptic weight *J* was included as a fourth uncertain parameter (with a similar relative spread as for the other uncertain parameters in Table 4). Here we observed that at least *p* = 7 (using the default number of collocation nodes) was needed to obtain accurate results. This illustrates that the required polynomial order, and by extension the required number of samples *N*_*s*_, to get accurate results is problem dependent.

#### 4.4.1. Average interspike interval

**Figure 8:**
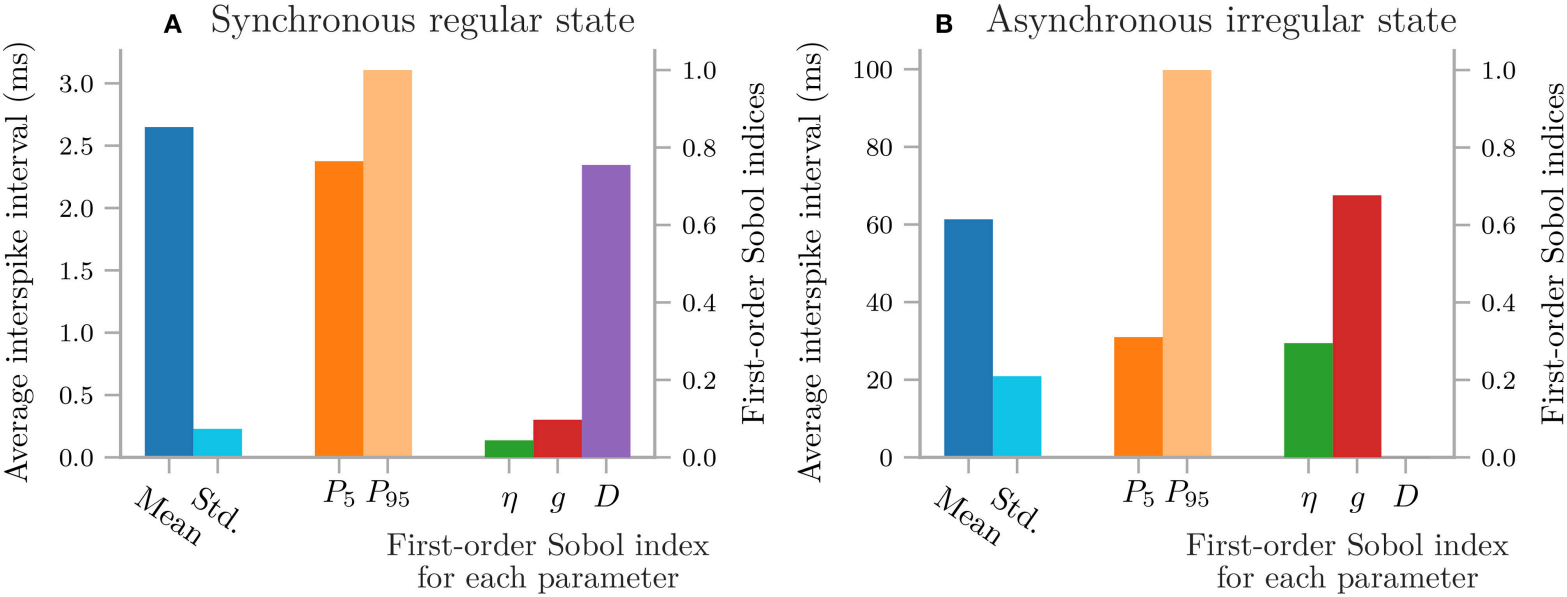
The average interspike interval for the Brunel network in the two states. Mean, standard deviation, 90% prediction interval, and first-order Sobol indices of the average interspike interval of the Brunel network in the synchronous regular state **(A)**, and asynchronous irregular state **(B)**. The 90% prediction interval is indicated by the 5th and 95th percentiles, i.e., 90% of the average spike intervals are between *P*_5_ and *P*_95_.

The average interspike interval is the average time it takes from a neuron produces a spike until it produces the next spike, averaged over all recorded neurons. The uncertainty quantification and sensitivity analysis of the average interspike interval of the Brunel network are shown in Figure 8. The average interspike interval is seen to differ strongly between the SR and AI states. In the high-firing SR state (Figure 8A), the mean of the average interspike interval is low, with a comparatively low standard deviation reflecting the synchronous firing in the network. We can observe this in Figure 7A, where the interspike intervals are short and do not vary much (i.e., very little standard deviation). In the comparatively low-firing AI state (Figure 8B), the mean of the average interspike interval is high, with a large standard deviation, reflecting the irregular firing in the network seen in Figure 7B.

The two states were also found to be different in terms of which parameters the average interspike interval is sensitive to. In the SR state the network is predominantly sensitive to the synaptic delay *D*. This reflects that in this state the neurons get very strong synaptic inputs so that the firing rate is mainly determined by the delay. In the AI state, the network is more balanced and “variance-driven”, and the dynamics are to a large degree determined by the relative strength of the inhibitory synapses compared to the excitatory synapses *g* (Brunel, 2000). Thus the average interspike interval is observed in Figure 7B to, not surprisingly, be most sensitive to *g*. In the AI state the average interspike interval is quite long (~60 ms) so that an uncertainty in the synaptic delay of a couple of milliseconds (cf. Table 4) has little influence. Thus very little sensitivity to *D* is observed in this state.

#### 4.4.2. Correlation coefficient

**Figure 9:**
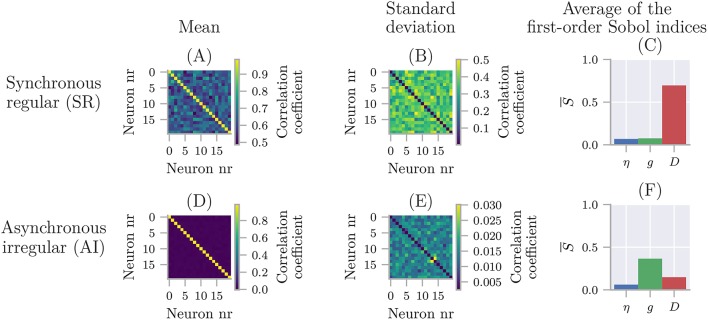
The pairwise Pearson's correlation coefficient for the Brunel network in the two states. Mean **(A,D)**, standard deviation **(B,E)**, and first-order Sobol indices **(C,F)** for the pairwise Pearson's correlation coefficient of the Brunel network in the synchronous regular **(A–C)** and asynchronous irregular **(D–F)** states.

The pairwise Pearson's correlation coefficient is a measure of how synchronous the spiking of a network is. This correlation coefficient measures the correlation between the spike trains of two neurons in the network. In Figure 9 we examine how this correlation depends on parameter uncertainties by plotting the mean, standard deviation, and first-order Sobol indices for the pairwise Pearson's correlation coefficient in the SR and AI states.

As expected from examining Figure 7, the mean pairwise Pearson's correlation coefficient in the SR state (Figure 9A) is much higher than in the AI state (Figure 9D). The first-order Sobol indices further show that the degree of synchronicity is by far most sensitive to the synaptic delay *D* when the network is in the SR state (Figure 9C), and most sensitive to the relative strength of inhibitory synapses *g* when the network is in the AI state (Figure 9F).

Thus, for both features investigated here (the average interspike interval and the mean pairwise Pearson's correlation coefficient), the conclusions regarding model sensitivity are the same. The SR state of the Brunel network is most sensitive to the synaptic delay *D*, while the AI state is most sensitive to the relative strength of inhibitory synapses *g*.

### 4.5. Comparing the quasi-Monte Carlo method to polynomial chaos expansions

To compare the efficiency of the polynomial chaos expansions and the quasi-Monte Carlo method, we calculate the errors of the uncertainty quantification for the Hodgkin-Huxley model (section 4.2) using a varying number of model evaluations. The code for this comparison can be found in uncertainpy/examples/mc_vs_pc.

As efficiency measure we use the number of model evaluations *N*_*s*_, since model evaluation generally is the computationally most costly step. We examine two versions of the Hodgkin-Huxley model to see how the efficiency of the two methods varies with the number of uncertain parameters. We use a reduced model with the three maximum conductances ḡ_Na_, ḡ_K_, and ḡ_L_ as uncertain parameters, and a complete model where all eleven parameters are uncertain. As in section 4.2, we assume each of these parameters to have a uniform distribution in the range ±10% around their original value. We use polynomial chaos expansions with the point-collocation method, where the number of evaluations equals the number of collocation nodes.

As error measure we use the average of the absolute relative error over time, which we simply will refer to as the error:

(22) $$\begin{array}{l} \varepsilon_{X}=\frac{1}{T}\int \frac{\left|X-X_{\text{estimate}}\right|}{X}\text{d}t, \end{array}$$

where “estimate” indicates the results from either the quasi-Monte Carlo method or the polynomial chaos expansions. *T* is the total simulation time in the model, disregarding the first 5 ms. *X* is either the mean 𝔼[*Y*], variance 𝕍[*Y*], or first-order Sobol indices *S*_*i*_ averaged over all parameters *i*.

Since an analytical solution for the Hodgkin-Huxley model is not available, we use the quasi-Monte Carlo method with 200,000 model evaluations to calculate the “exact” 𝔼[*Y*] and 𝕍[*Y*], and 100000(*d* + 2) (where *d* is the number of uncertain parameters) model evaluations to calculate *S*_*i*_. The quasi-Monte Carlo method is based on random sampling, so we calculate the average error of 50 re-runs for the quasi-Monte Carlo method, to get a more precise result.

**Figure 10:**
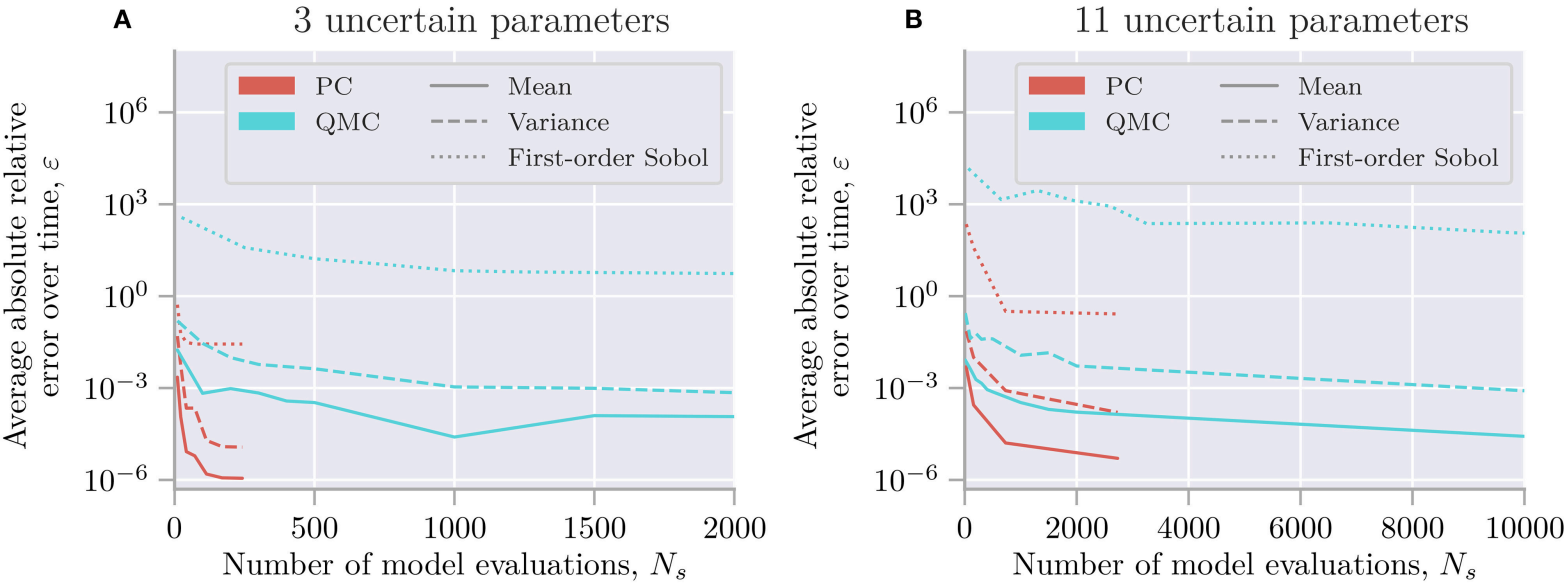
The error of the mean, variance and (average) first-order Sobol indices for the quasi-Monte Carlo method (QMC) and polynomial chaos expansions (PC) used on the Hodgkin-Huxley model. The average of the absolute relative error over time of the mean (Equation 3), variance (Equation 4), and first-order Sobol indices (Equation 7) (averaged over all parameters *i*) of the Hodgkin-Huxley model with three **(A)** and eleven **(B)** uncertain parameters. The mean, variance and first-order Sobol indices are calculated using the quasi-Monte Carlo method with 50 re-runs, and polynomial chaos expansion with point collocation. The “exact” solutions are found using the quasi-Monte Carlo method with *N*_*s*_ = 200000 model evaluations to calculate the mean and variance, and *N*_*s*_ = 100000(*d* + 2) model evaluations (where *d* is the number of uncertain parameters) to calculate the Sobol indices.

The error of the mean, variance, and first-order Sobol indices of the two methods for the two variants of the model are shown in Figure 10. We clearly see that the polynomial chaos expansions are much faster than the quasi-Monte Carlo method for both test cases, that is, much fewer model evaluations *N*_*s*_ are needed to achieve a certain error.

Figure 10 shows the error for the Hodgkin-Huxley model with three uncertain parameters. In this case, the quasi-Monte Carlo method requires more than 200 times as many model evaluations as the polynomial chaos expansions to calculate the mean with an error of ~ 10^−5^, and more than 2,500 times as many model evaluations to calculate the Sobol indices with an error of ~ 0.5.

Figure 10B shows the error for the Hodgkin-Huxley model with eleven uncertain parameters. By comparing with the results for three uncertain parameters, we observe that polynomial chaos expansions scale worse with the number of uncertain parameters than the quasi-Monte Carlo method. However, polynomial chaos expansions are still superior in regards to the required number of model evaluations. For the full Hodgkin-Huxley model, the quasi-Monte Carlo method needs more than ten times as many model evaluations as the polynomial chaos expansions to calculate the mean with an error of ~ 2 · 10^−5^. For the first-order Sobol indices the quasi-Monte Carlo method gives an error of more than 30 even after 65, 000 evaluations. In contrast, the polynomial chaos expansions give an error of 0.26 after only 2, 732 model evaluations.

### 4.6. Additional examples

Additional examples for uncertainty quantification of the Izikevich neuron (Izhikevich, 2003), a reduced layer 5 pyramidal cell (Bahl et al., 2012), and a Hodgkin-Huxley model with shifted voltage (Sterratt et al., 2011) are found in uncertainpy/examples/.

## 5. Discussion

A major challenge with models in neuroscience is that they tend to contain several uncertain parameters whose values are critical for the model behavior. In this paper we have presented Uncertainpy, a Python toolbox which quantifies how uncertainty in model parameters translates into uncertainty in the model output and how sensitive the model output is to changes in individual model parameters. Uncertainpy is tailored for neuroscience applications by its built-in capability for recognizing features in the model output.

The key aim of Uncertainpy is to make it quick and easy for the user to get started with uncertainty quantification and sensitivity analysis, without any need for detailed prior knowledge of uncertainty analysis. Uncertainpy is applicable to a wide range of different model types, as illustrated in the example applications. These included an uncertainty quantification and sensitivity analysis of four different models: a simple cooling coffee-cup model (section 4.1), the original Hodgkin-Huxley model for generation of action potentials (section 4.2), a multi-compartmental NEURON model of a thalamic interneuron (section 4.3), and a NEST model of a sparsely connected recurrent (Brunel) network of integrate-and-fire neurons (section 4.4). These analyses were mainly performed to illustrate the use of Uncertainpy, but also revealed both expected and unexpected features of the example models. However, we did not put any effort into estimating realistic distributions for the parameter uncertainties. The conclusions should therefore be treated with caution; see result sections for a detailed discussion.

To our knowledge, Uncertainpy is the first toolbox to use polynomial chaos expansions to perform uncertainty quantification and sensitivity analysis in neuroscience. Compared to the (quasi-)Monte Carlo method, polynomial chaos expansions dramatically reduce the number of model evaluations needed to get reliable statistics when the number of uncertain parameters is relatively low, typically smaller than about 20 (Xiu and Hesthaven, 2005; Crestaux et al., 2009; Eck et al., 2016). This was also observed in the present study where we in section 4.5 found that polynomial chaos expansions require one to three orders of magnitude fewer model evaluations than the quasi-Monte Carlo method when applied to the Hodgkin-Huxley model with three or eleven uncertain parameters. This gain in efficiency is especially important for models that require a long simulation time, where uncertainty quantification using the (quasi-)Monte Carlo method could require an unfeasible amount of computer time.

### 5.1. Application of uncertainpy

Uncertainpy is a computationally efficient Python toolbox that enables uncertainty quantification and sensitivity analysis for computational models. It is tailored toward neuroscience applications by its built-in capability for calculating characteristic features of the model output. While Uncertainpy has a broad applicability, as demonstrated in this paper, certain limitations exist. The first, and perhaps most obvious, is that Uncertainpy does not deal with the problem of obtaining the distributions of the uncertain parameters.

It is also typically not obvious which model is best suited to describe a particular system. For example, when we construct a neural model we first have to decide which mechanisms (ion channels, ion pumps, synapses, network connectivity, etc.) to include in the model. Next, we select a set of mathematical equations that describe these mechanisms. Such choices are seldom trivial, and no methods for resolving this structural uncertainty aspect of modeling are included in Uncertainpy. Nevertheless, quantitative measures such as those obtained with Uncertainpy may still give valuable insight in the relationship between model parameters and model output, which can guide experimentalists toward focusing on accurately measuring the parameters most critical for the model output. Additionally, it can guide modelers by identifying mechanisms that can be sacrificed for model reduction purposes.

The accuracy of the quasi-Monte Carlo method and polynomial chaos expansions is problem dependent and is determined by the number of samples, as well as the polynomial order for polynomial chaos expansions. It is therefore a good practice to examine if the results from the uncertainty quantification and sensitivity analysis have converged (Eck et al., 2016). A simple method for checking the convergence is to change the number of samples or polynomial order, or both, and examine the differences between the results. We can be reasonably certain that the results are accurate once these differences are small enough.

### 5.2. Further development of uncertainpy

There are several ways that Uncertainpy can be further developed. If a model or features of a model are irregular, Uncertainpy performs an interpolation of the output to get the results on the regular form needed in the uncertainty quantification and sensitivity analysis. Currently, Uncertainpy only has support for interpolation of one-dimensional output (vectors), but this aspect can be improved.

The screening method available in Uncertainpy is unable to take interactions between parameters into account. More advanced screening methods able to do this exist (Morris, 1991; Campolongo et al., 2007) and could be implemented.

The built-in feature library in Uncertainpy can easily be expanded by adding additional features. The number of built-in simulators (at present NEST and NEURON) can also easily be extended. We encourage the users to add custom features and models through Github pull requests.

### 5.3. Outlook

In many fields of the physical sciences, the model parameters that go into simulations are known with high accuracy. For example, in quantum mechanical simulations of molecular systems, the masses of the nuclei and electrons, as well as the parameters describing their electrical interaction, are known so precisely that uncertainty in model parameters is not an issue (Marx and Hutter, 2009). This is not the case in computational biology in general, and in computational neuroscience in particular. Model parameters of biological systems often have an inherent variability and some may even be actively regulated and change with time. They can therefore not be precisely known. We thus consider uncertainty quantification and sensitivity analysis to be particularly important in computational biology.

Uncertainpy was developed with the aim of enabling such analysis, that is, to provide an easy-to-use tool for precise evaluation of the effect of uncertain model parameters on model predictions. Being an open-source Python toolbox, we hope that Uncertainpy can be further developed through a joint effort within the neuroscience community.

## Author contributions

ST, GH, and GE conceived of and designed the project. ST designed, wrote, tested, and documented the software and performed analysis of the examples. ST, GH, and GE wrote and revised the paper.

### Conflict of interest statement

The authors declare that the research was conducted in the absence of any commercial or financial relationships that could be construed as a potential conflict of interest.

## Footnotes

^1^https://github.com/simetenn/uncertainpy

^2^https://github.com/SALib/SALib/issues/134

^3^http://uncertainpy.readthedocs.io/

^4^http://uncertainpy.readthedocs.io/

^5^http://efel.readthedocs.io

^6^https://github.com/SALib/SALib/issues/134

^7^https://www.docker.com/

^8^https://zenodo.org/
